# To New Beginnings: Riboproteogenomics Discovery of N-Terminal Proteoforms in *Arabidopsis Thaliana*

**DOI:** 10.3389/fpls.2021.778804

**Published:** 2022-01-06

**Authors:** Patrick Willems, Elvis Ndah, Veronique Jonckheere, Frank Van Breusegem, Petra Van Damme

**Affiliations:** ^1^Department of Plant Biotechnology and Bioinformatics, Ghent University, Ghent, Belgium; ^2^Vlaams Instituut voor Biotechnologie (VIB)-Center for Plant Systems Biology, Ghent, Belgium; ^3^integrative Riboproteogenomics, Interactomics and Proteomics Unit, Laboratory of Microbiology, Department of Biochemistry and Microbiology, Ghent University, Ghent, Belgium

**Keywords:** translation initiation site, ribosome profiling, N-terminal proteomics, N-terminal acetylation, *Arabidopsis thaliana*, alternative translation initiation, chloroplast transit peptide, riboproteogenomics

## Abstract

Alternative translation initiation is a widespread event in biology that can shape multiple protein forms or proteoforms from a single gene. However, the respective contribution of alternative translation to protein complexity remains largely enigmatic. By complementary ribosome profiling and N-terminal proteomics (i.e., riboproteogenomics), we provide clear-cut evidence for ~90 N-terminal proteoform pairs shaped by (alternative) translation initiation in *Arabidopsis thaliana*. Next to several cases additionally confirmed by directed mutagenesis, identified alternative protein N-termini follow the enzymatic rules of co-translational N-terminal protein acetylation and initiator methionine removal. In contrast to other eukaryotic models, N-terminal acetylation in plants cannot generally be considered as a proxy of translation initiation because of its posttranslational occurrence on mature proteolytic neo-termini (N-termini) localized in the chloroplast stroma. Quantification of N-terminal acetylation revealed differing co- vs. posttranslational N-terminal acetylation patterns. Intriguingly, our data additionally hints to alternative translation initiation serving as a common mechanism to supply protein copies in multiple cellular compartments, as alternative translation sites are often in close proximity to cleavage sites of N-terminal transit sequences of nuclear-encoded chloroplastic and mitochondrial proteins. Overall, riboproteogenomics screening enables the identification of (differential localized) N-terminal proteoforms raised upon alternative translation.

## Introduction

Translation is a vital and tightly controlled cellular process involving translation initiation, elongation, termination, and ribosome recycling phases. Of these phases, translation initiation forms a major rate-limiting step that is strongly regulated in eukaryotes (Sonenberg and Hinnebusch, 2009; Shah et al., 2013). Efficient translation initiation typically requires a start codon to be embedded in a specific sequence context known as the Kozak motif (Kozak, 1986). However, the prediction of protein start sites is challenged by the occurrence of translation initiation in non-AUG codons, leaky scanning, and internal ribosome entry sites (IRES) among others (Ingolia et al., 2011). Study on translation as well as translation initiation has been revolutionized by next-generation sequencing methods that analyze ribosomal occupancy. Using chemical inhibitors that halt ribosomes during translation initiation [e.g., lactimidomycin (LTM) (Lee et al., 2012)] or elongation phases [typically cycloheximide (CHX) (Ingolia et al., 2011; Lee et al., 2012)], ribosome-protected mRNA fragments can be isolated and sequenced, a method designated ribosome profiling or Ribo-seq (Lee et al., 2012). Also, for the model plants *Arabidopsis* (*Arabidopsis thaliana*) and tomato (*Solanum lycopersicum*), translation (initiation) landscapes have been mapped by means of Ribo-seq (Liu et al., 2013; Willems et al., 2017; Li and Liu, 2020). Strikingly, these studies revealed that at least half of the identified translation initiation sites (TIS) are miss- or unannotated, demonstrating the widespread occurrence of alternative TIS (aTIS) further increasing proteome complexity (Li and Liu, 2020). For instance, aTIS can give rise to distinct proteoforms encoded in-frame from a single gene (i.e., N-terminal extended or truncated proteoforms) that have specialized functions or display differences in subcellular localization of stability (Gawron et al., 2016; Fijalkowska et al., 2020; Li and Liu, 2020; Jonckheere and Van Damme, 2021). However, protein-level evidence of multiple proteoforms synthesized from such alternative TIS events is still in its infancy in plants.

Positional proteomics, and more specifically N-terminal proteomics, may offer orthogonal evidence of initiation of protein synthesis. In a previous study, we have enriched and identified N-terminal (Nt) peptides in *Arabidopsis* cell cultures by Nt Combined FRActional Diagonal Chromatography (COFRADIC) (Staes et al., 2011; Willems et al., 2017). Intriguingly, several identified Nt peptides matched Ribo-seq TIS in intergenic regions of the *Arabidopsis* genome, showing thus great promise to identify novel protein-coding genes (Willems et al., 2017). Protein N-termini frequently lack the initiator Met (iMet) and are subject to Nt acetylation (NTA) because of the consecutive co-translational actions of Met aminopeptidases (MetAPs) (Frottin et al., 2006; Jonckheere et al., 2018) and N-acetyltransferases (NATs) (Bienvenut et al., 2012) on nascent polypeptides. Next to Nt peptides matching TIS, protein N-termini can result from proteolytic processing of proteins (i.e., neo-N-termini). Intriguingly, in plants, NTA also occurs post-translationally on stromal protein N-termini generated upon removal of the chloroplast transit peptide (cTP) by the stromal processing peptidase (SPP) and possible further trimming by aminopeptidases (Wirtz et al., 2010; Rowland et al., 2015). In this study, riboproteogenomic screening revealed compelling evidence for the synthesis of multiple Nt proteoforms shaped by (alternative) translation initiation originating from 68 *Arabidopsis* genes with potentially diversifying functions and/or subcellular locations. More so, half of the coding sequences encoding such Nt proteoform pairs are predicted to shape cytosolic protein copies next to encoding plastid or mitochondrion-localized Nt proteoforms.

## Materials and Methods

### Ribo-Seq Analysis

#### Genome Alignment and Ribosome Footprints

Ribosome profiling library generation and sequencing of *Arabidopsis* cell suspension cultures have been described previously (Willems et al., 2017). For Ribo-seq data analysis, reads were processed using the PROTEOFORMER pipeline (Crappe et al., 2015). The reads were trimmed for adaptor sequences using fastx_clipper (version 0.0.14) and subsequently aligned to the *Arabidopsis* rRNA or tRNA allowing for two mismatches using STAR (version 2.5.1b) (Dobin et al., 2013). Reads aligning to these indices were discarded, and unmapped reads were aligned onto the *Arabidopsis* TAIR10 genome, also allowing for reads with, at most, two mismatches and mapping to a maximum of 16 locations within the genome. These tolerant-matching parameters were selected based on higher peptide identification rates of annotated proteins in the resulting Ribo-seq-based protein database (Crappe et al., 2015). P-site positioning of the ribosome was determined by using only footprints of length 26–34 bp. An offset of +12, +13, and +14 from the 5′ end of the reads was used for reads of lengths 26–30, 31–33, and 34 bp, respectively. The positional scores represent the number of read alignment attributed to each genomic position.

#### Translation Initiation Site Calling

The mapped profiles from the initiating ribosomes, obtained after lactimidomycin (LTM) treatment, were found accumulated in AUG or near-cognate start codons using a ±1 nt window (Ingolia et al., 2011; Lee et al., 2012). Profiles that did not map within this window relative to the first position of an assigned start codon were discarded. Furthermore, accumulation peak positions had to comply with a number of criteria in order to be withheld as a true TIS (Lee et al., 2012): (i) the identified TIS should have the maximal number of LTM reads within a window of seven nucleotides (i.e., 3 nt up- and down-stream of the P-site assignment), (ii) the combined number of ribosome footprints for the TIS should exceed a minimal profile count threshold (see below), and (iii) the TIS should have a difference in normalized reads between LTM-treated and CHX-treated samples (*R*_*LTM*_ − *R*_*CHX*_) equal or higher than a certain threshold, where:


$$\begin{array}{l} R_{k}=\left(\frac{X_{k}}{N_{k}}\right)\times 10\;\left(k=LTM\;or\;CHX\right)\\ X_{k}=number\;of\;reads\;on\;position\;X\;for\;data\;k\\ N_{k}=total\;number\;of\;reads\;on\;transcript\;for\;data\;k \end{array}$$

This *R*_*LTM*_−*R*_*CHX*_ represents a useful feature to discern translated TIS, with higher values indicating stronger translation initiation (LTM) read coverage compared to elongation (CHX) read coverage. The thresholds for TIS “minimum profile count” and *R*_*LTM*_ −*R*_*CHX*_ were optimized for database annotated [Araport11 (Cheng et al., 2017) or TAIR10] TIS (dbTIS) and selected based on the impact on the peptide identification rate in the matching proteomics datasets. We opted for a categorized approach based on TIS localization: for dbTIS the abovementioned thresholds were set to five counts and a *R*_*LTM*_ − *R*_*CHX*_ ≥ 0.01, respectively. dbTIS that did not comply with the aforementioned criteria were also taken into account if the annotated coding sequence (CDS) showed elongating ribosome occupancy. For other TIS categories, more stringent threshold settings were used in order to limit the number of false-positive Ribo-seq called TIS. More specifically, the thresholds for a TIS located in 5′ and 3′ leader sequences, or intergenic regions were set to 10 counts and a *R*_*LTM*_ − *R*_*CHX*_ ≥ 0.05, while for a TIS located in the CDS downstream of a dbTIS, the thresholds were set to 15 counts and a *R*_*LTM*_ − *R*_*CHX*_ ≥ 0.15, respectively. TIS that were non-compliant with these rules were discarded. Overall, applying these settings resulted in a total of 29,013 Ribo-seq called TIS (Supplementary Dataset S1).

#### Normalized Ribosome Footprint Density Plot

Ribosome footprints (RPFs) were normalized by dividing RPF counts by the average RPF counts over the CDS and 20 positions upstream and downstream in the respective leader sequences. CHX- and LTM-normalized densities were plotted for 14,220 and 13,421 genes, respectively, requiring at least 20 mapped CHX/LTM RPFs per gene.

### N-Terminal Proteomics—*Arabidopsis* Cell Suspension Cultures

Raw Nt proteomics data of *Arabidopsis* cell cultures acquired previously (Willems et al., 2017) were re-analyzed for comprehensive identification of Nt proteoforms. First, RAW files were converted to peak lists [Mascot Generic Format (MGF) files] using ThermoRawFileParser (Hulstaert et al., 2020). Resulting peak lists were searched with COMET (Eng et al., 2013) against a custom protein database (available at the Open Science Framework project “ajx5e” [https://osf.io/ajx5e/]) consisting of TAIR10 protein entries (such as splice forms) appended with novel proteoform sequences initiated from 15,741 Ribo-seq-called TIS not annotated in TAIR10 (Supplementary Dataset S1). It should be noted that 52 of these TIS were, however, annotated in the more recent Araport11 re-annotation (Cheng et al., 2017). Variable modifications for database searching included Nt light/heavy acetylation (+42.011 or 47.036 Da, respectively) and pyro-Glu formation from Gln (−17.026 Da). Fixed modifications were Met oxidation (+15.995 Da), Lys heavy acetylation (+47.036 Da), and Cys carbamidomethylation (+57.021 Da). For the respective datasets, Trypsin, Glu-C, Asp-N, and chymotrypsin were set as a digestive enzyme, specifying two missed cleavages for trypsin and three missed cleavages for all other enzymes. For the searches, no enzymatic digestion rules apply to the N-terminus of the searched peptides (Comet option “num_enzyme_termini = 9”) to additionally enable the identification of database non-annotated Nt peptides. Percolator output files were processed by Percolator (version 3.05.0) (The et al., 2016) using the options “–Y –trainFDR 0.05 –testFDR 0.05” and resulting Nt peptide spectral identifications (PSM q-value ≤ 0.01) were parsed. In the overview list of protein N-termini reported (Supplementary Dataset S2), Nt peptides with an identical protein start position but varying at their C-termini because of digestion by different enzymes or missed cleavages were collapsed to the longest Nt peptide variant, thereby reducing redundancy and improving the uniqueness of peptide-to-protein assignments.

### N-Terminal Proteomics—Chloroplast—and Stromal-Enriched Fractions of Arabidopsis Leaves

#### Plant Materials and Growth Conditions

For the Nt proteomics analysis of the proteome content of enriched chloroplasts and stroma, *Arabidopsis* wild-type plants (ecotype Columbia-0) were grown at 21°C under short-day conditions (8-/16-h photoperiod) in a half-strength Murashige and Skoog medium (Duchefa, Haarlem, The Netherlands) containing 0.8% w/v sucrose. Intact chloroplasts and a fraction enriched for the stromal protein content were isolated from 4-week-old plant leaves as described in Block et al. (2002), and single fractionation was performed to enrich for chloroplasts and chloroplast stroma. More specifically, the chloroplasts were harvested from the green interphase of a two-step Percoll gradient, and the stromal fraction was enriched by chloroplast breakage through resuspension of the chloroplast-enriched pellet in a 4-mM MgCl_2_ containing 10 mM MOPS (pH 7.6) buffer followed by top layer collection after sucrose gradient centrifugation.

#### N-Terminal COFRADIC and LC-MS/MS Analyses

Nt COFRADIC was essentially performed as described previously (Willems et al., 2017). The pellet enriched for intact chloroplasts were immediately resuspended in an ice-cold buffer [50 mm sodium phosphate, pH 7.5, 100 mm NaCl and 1 × cOmplete™, and EDTA-free protease inhibitor mixture (Roche, Basel, Switzerland)], left on ice for 10 min, and subjected to 3 freeze-thaw cycles. Supernatant was recovered by centrifugation at 16,000 × *g* for 15 min at 4°C. In the case of stromal proteome, an additional buffer exchange (4 M guanidinium hydrochloride, 50 mM sodium phosphate, pH 7.5) of the top layer collected after sucrose gradient centrifugation was performed by making use of a Sephadex G-25 size-exclusion desalting column (PD-10, cat n°17-0851-01; GE Healthcare Bio-Sciences, Chicago, IL, United States).

In the case of isolated proteomes of enriched chloroplast or stromal fractions, two Nt COFRADIC proteome analyses were performed in parallel. More specifically, primary free amine modification of the isolated proteomes was either *in vitro N*-acetylated using an *N*-hydroxysuccinimide ester of ^13^C_2_D_3_-acetate (heavy *N*-acetylation) or left unmodified. While *in vitro* heavy *N*-acetylation allows distinguishing between natural and *in vitro* NTA, it additionally allows assessing the degree or stoichiometry of NTA (Van Damme et al., 2011). Otherwise, omitting the *in vitro N*-acetylation step renders Lys susceptible to trypsin cleavage (otherwise blocked by acetylation) and could, thus, provide complementary Nt peptide evidence. Subsequent steps of the Nt COFRADIC procedure were performed as described previously (Willems et al., 2017). Reverse phase high-performance liquid chromatography (RP-HPLC) fractions enriched for protein N-termini were all introduced into the Ultimate 3000 (Dionex, Amsterdam, The Netherlands) in-line connected to an LTQ Orbitrap XL mass spectrometer (Thermo Fisher Scientific), and liquid chromatography tandem mass spectrometry (LC-MS/MS) analysis was performed as described previously (Willems et al., 2017).

#### Peptide Identification and Quantification of the Degree of Nt-Acetylation

Peak lists (Mascot Generic Format [MGF] files) were created from raw proteomic files using the Mascot Distiller software (version 2.3.2.0; Matrix Science, Boston, MA, United States). Where possible, grouping of spectra with 0.005 Da precursor tolerance was allowed with a maximum intermediate retention time of 30 s and a maximum intermediate scan count of five. There was no de-isotoping, and the relative signal-to-noise limit was set at two. These peak lists were then searched with the Mascot search engine (version 2.3; Matrix Science, Boston, MA, United States). Spectra were searched against the *Arabidopsis* TAIR10 proteome. In the case of plant proteomes labeled with heavy acetyl isotopes, Lys heavy acetylation (+47.036 Da), Cys carbamidomethylation (+57.021 Da), and Met oxidation (+15.995 Da) were set as fixed modifications. Variable modifications included Nt light/heavy acetylation (+42.011 or 47.036 Da) and pyro-Glu formation from Gln (−17.026 Da). In case no heavy acetyl labeling step was performed, no fixed Lys modification or variable Nt modification was searched. Mass tolerance on precursor ions was set to 10 ppm (with Mascot's C13 option set to 1) and on fragment ions to 0.5 Da. Endoproteinase semi-Arg-C/P (Arg-C specificity with Arg-Pro cleavage allowed) was set as enzyme in case of heavy *N*-acetylated samples (modified Lys), whereas trypsin/P specificity was selected in case of non-heavy *N*-acetylation samples; both allowing no missed cleavages. The peptide charge was set to 1+, 2+, and 3+, and instrument setting was put to ESI-TRAP. Only Nt peptides that were ranked one and scored above the threshold score, set at 99% confidence, have a minimum amino acid length of seven, and compliant with the rules of NTA or initiator methionine (iMet) processing were withheld (Helsens et al., 2011). Quantification of the degree of NTA was performed as described previously (Van Damme et al., 2011). All data management was performed in ms_lims (Helsens et al., 2010).

### Generation of TIS-Mutagenized CDSs by Site-Directed Mutagenesis PCR Coupled With *in vitro* Transcription and Translation

pUNI51 vectors (Arabidopsis Biological Resource Center [ABRC], Columbus, OH, United States) encoding full-length isopentenyl diphosphate isomerase 2 (IPP2, AT3G02780, stock number: U22155), plant UBX domain-containing protein 1 (PUX1, AT3G27310, stock number: U82310), and NAC domain-containing protein 14 (NAC014, AT1G33060, stock number: U88651) served as templates for site-directed PCR mutagenesis (QuickChange; Stratagene, San Diego, CA, United States). For cloning and propagation of pUNI51 vectors, the *Escherichia coli* (*E. coli*) strain PIR1 (One Shot^TM^ PIR1 chemically competent *E. coli*, cat n°C101010; Invitrogen, Waltham, MA, United States) was used using standard chemical transformation protocols (i.e., pUNI51 contains a conditional origin of replication derived from the R6Kγ plasmid that enables propagation only in bacterial hosts expressing the *pir* gene encoding the essential replication protein p) and with selection in the presence of 50 μg/ml kanamycin. Site-directed PCR mutagenesis was performed according to the manufacturer's instructions and using the primer pairs indicated in Supplementary Table 1 to mutate the riboproteogenomics-identified ATG start codons to the Leu codon TTG. The correctness of all (mutant) cDNA insert sequences was confirmed by Sanger sequencing using the pUNI51 forward and reverse primers 5′-CTGTTGGTGTGTCTATTAAATCG-3′ (pUNI51-fwd) and 5′-TGGCTGGCAACTAGAAGGCAC-3′ (pUNI51-rev), respectively.

To inspect translation products, the mutagenized constructs served as templates for *in vitro* coupled transcription/translation using a rabbit reticulocyte lysate system according to the manufacturer's instructions (TnT T3 Coupled Transcription/Translation Lysate System; Promega, Madison, WI, United States). More specifically, [^35^S] methionine-labeled translation products were generated using the TnT T3 RNA polymerase and 2 μl of [^35^S] methionine (10 μCi/ml) per reaction. After 1 h of incubation at 30°C, and to stop the translation reaction, 4 μl of the reaction mixture (50 μl reaction mixture in total) was diluted 12.5-fold in 50 mM Tris (pH 8) and NuPAGE^®^ LDS Sample Buffer (Invitrogen, Waltham, MA, United States), and the samples were heated for 10 min at 70°C. The samples were separated on 4–12% or 12% NuPAGE^®^ Bis-Tris gradient gels (1 mm × 12 well; Invitrogen, Waltham, MA, United States) using MOPS Buffer. Subsequently, proteins were transferred into a PVDF membrane, air-dried, and exposed to a film suitable for radiographic detection (ECL Hyperfilms; Amersham Biosciences, Buckinghamshire, United Kingdom).

### Bioinformatic Data Analysis

The stand-alone version of TargetP 2.0 (Almagro Armenteros et al., 2019) was used to predict cleavage sites of Nt sorting signals. iceLogo (Colaert et al., 2009) sequence motifs were generated on the online webserver (https://iomics.ugent.be/icelogoserver) using the precompiled Swiss-Prot composition of *Arabidopsis* as reference set. Nt peptide data stored in the Plant PTM Viewer (Willems et al., 2019) and NTerDB (https://n-terdb.i2bc.paris-saclay.fr/) were cross-referenced to the Ribo-seq-called TIS data. Additionally, proteins were appointed a subcellular location based on SUBA4 consensus locations (Hooper et al., 2017).

### Data Availability

All mass spectrometry proteomics data and search results of *Arabidopsis* cell cultures have previously been deposited to the ProteomeXchange Consortium *via* the PRIDE (Perez-Riverol et al., 2019) partner repository with the dataset identifier PXD004896 (Willems et al., 2017). Ribo-seq sequencing data have been deposited in NCBI's Gene Expression Omnibus and are accessible through GEO Series accession GSE88790. The PROTEOFORMER Ribo-seq MySQL database and its derived protein FASTA database and the mass spectrometry proteomics data corresponding to the Nt proteomics data of chloroplast and stromal enriched fractions of *Arabidopsis* leaves are all made available under the Open Science Framework project “ajx5e” (https://osf.io/ajx5e/).

## Results

### Mapping the Translation Initiation Landscape of *Arabidopsis*

**Figure 1:**
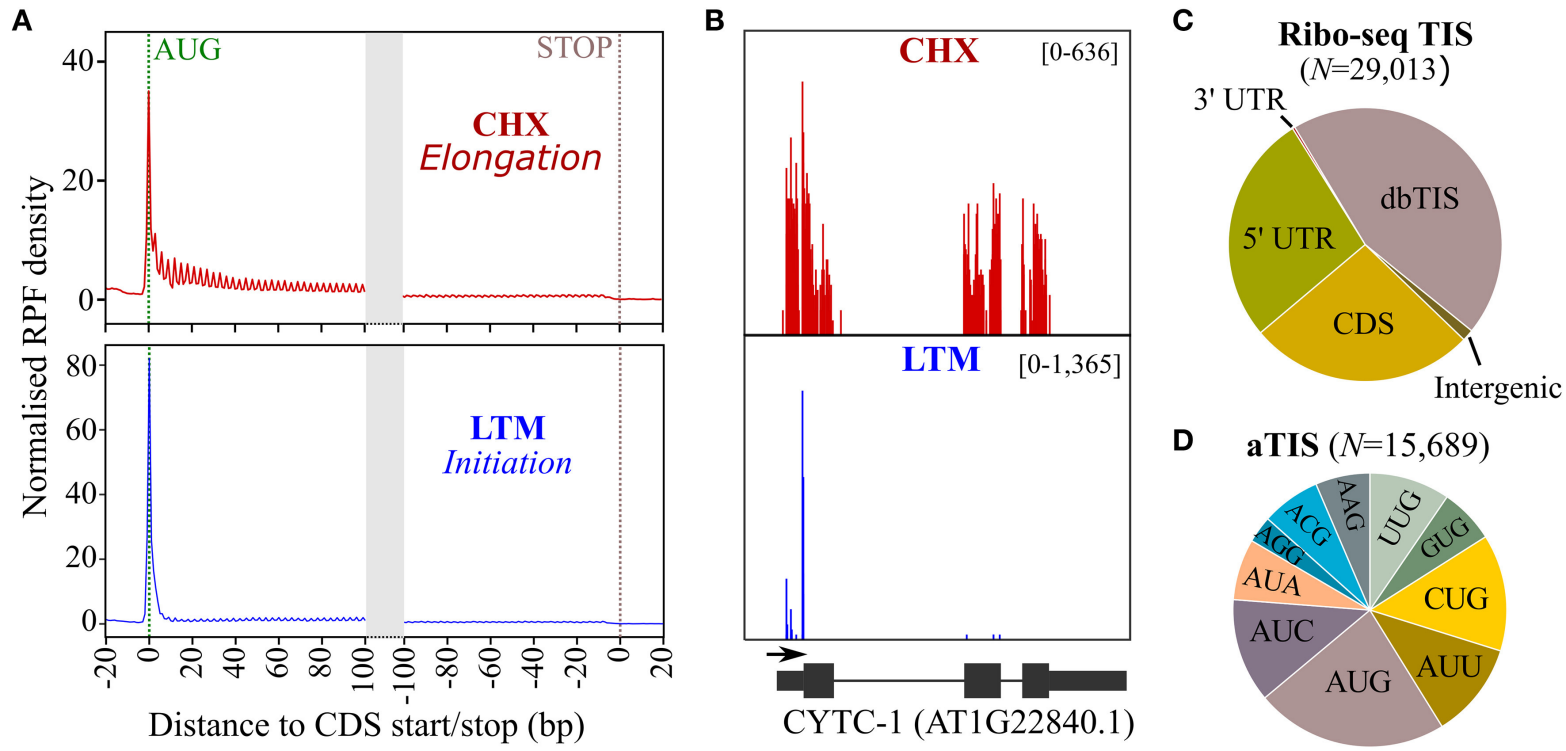
Ribosome sequencing (Ribo-seq) translation initiation site (TIS) calling in *Arabidopsis thaliana*. **(A)** Normalized ribosome footprint (RPF) density in TAIR10 *Arabidopsis* annotated coding sequence (CDS, representative gene model) regions after treatment with cycloheximide (CHX, red), which halts translation elongation, and lactimidomycin (LTM, blue), which halts translation initiation. **(B)** CHX (red) and LTM (blue) read coverage for cytochrome C-1 (AT1G22840.1). **(C)** Location of TIS called by PROTEOFORMER with respect to annotated TAIR10 gene models (for overview, see Supplementary Dataset S1). **(D)** Start codon distribution for called alternative TIS (aTIS).

We previously used Ribo-seq and Nt proteomics data in a complementary fashion for delineating unannotated protein-coding ORFs in intergenic regions of *Arabidopsis* (Willems et al., 2017). Using the translation inhibitors lactimidomycin (LTM) and cycloheximide (CHX), ribosomes were halted to determine ribosome occupancy during translation initiation and elongation, respectively, thereby providing complementary evidence for unannotated protein start sites. Here, we re-purposed the Ribo-seq data to discover alternative TIS (aTIS) within annotated protein-coding regions located downstream from database-annotated protein start sites (dbTIS) indicative of translation initiation events potentially giving rise to the expression of Nt proteoform pairs. As previously shown, a normalized distribution of ribosome footprints after CHX and LTM inhibition display strong signals at TAIR10-annotated starts and a triplet periodicity indicative of translation (Figure 1A). For instance, a strong LTM peak clearly and uniquely delineates the dbTIS of *Arabidopsis CYTOCHROME C-1*, while CHX coverage spans all three exonic regions (Figure 1B). Next, we used the high-quality CHX and LTM data (Figure 1A) for TIS calling in the *Arabidopsis* genome using PROTEOFORMER (Crappe et al., 2015) (see section Materials and methods). This resulted in the automated Ribo-seq-based calling of 29,013 TIS (Supplementary Dataset S1), including 13,324 TIS (46%) mappings to TAIR10/Araport11 dbTIS of 13,069 genes (Figure 1C). Besides the 71 TIS located in 3′ leader sequences (so called 3′ UTRs), 7,572 called TIS reside in the 5′ leader sequences (5′ UTRs) of 4,486 genes, corroborating the widespread occurrence of upstream ORF (uORF) translation events in *Arabidopsis* (Von Arnim et al., 2014; Niu et al., 2020). Interestingly, another 7,653 TIS were located in the CDSs of 2,818 genes, including possible downstream TIS (dTIS) that could potentially give rise to alternative, truncated Nt proteoforms in the case of in-frame dTIS. Considering an additional 393 TIS peaks residing outside of annotated genes, more than half of the called TIS (15,689 TIS) hint at database non-annotated TIS or alternative TIS (aTIS) (Figure 1C). These aTIS show substantial initiation at non-AUG, near cognate start codons (i.e., codons differing from AUG by a single nt). More specifically, besides 20.8% of AUG aTIS, initiation at near-cognate start codons, such as CUG (14.1%), AUC (12.6%), AUU (11.6%), and UUG (9.74%) was prevalent (Figure 1D). While a similar start codon usage of non-annotated TIS can be observed for the 5′ and 3′ leader sequences as well as the CDSs of annotated genes, a significant larger proportion of 169 AUG start codons (43%, χ^2^ test *p* < 0.001) was apparent among the 393 TIS identified in so-called intergenic regions (Supplementary Figure 1). This category of TIS may reflect the protein-coding potential of pseudogenes or transposable elements, besides un-annotated TIS upstream of dbTIS giving rise to Nt extended proteoforms or, alternatively, the discovery of novel gene products as reported previously (Willems et al., 2017). Taken together, Ribo-seq TIS calling mapped a wealth of unannotated (54%), besides annotated, TIS (15,689 vs. 13,324 TIS, respectively), suggesting a widespread unexplored TIS landscape in *Arabidopsis* (Figures 1C,D).

### N-Terminal Proteomics Provides Complementary Evidence for N-Terminally Truncated Proteoforms Shaped by Alternative Translation Initiation

**Figure 2:**
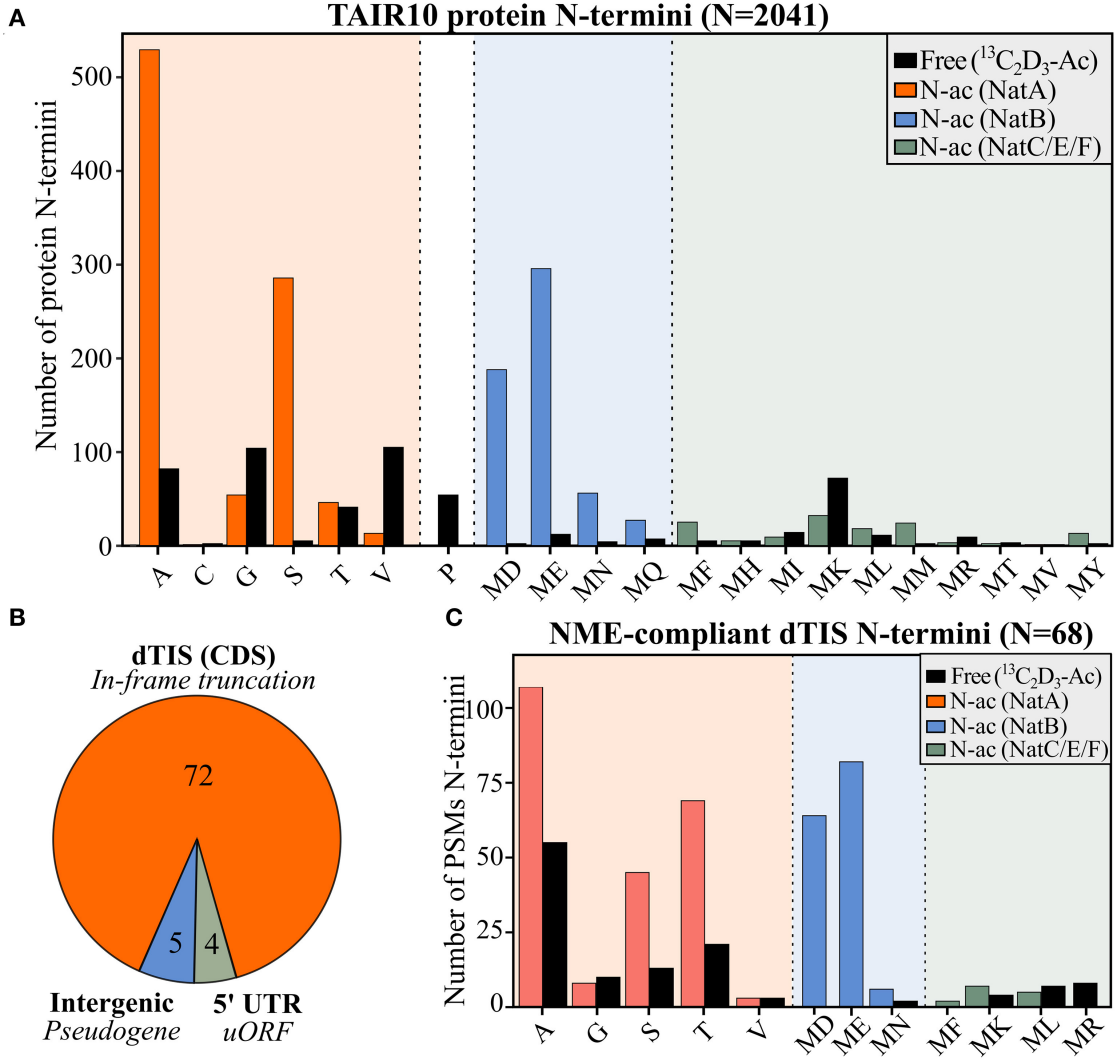
N-terminal peptide evidence for annotated and alternative TIS. **(A)** Number of identified Nt acetylation (NTA) or Nt-free (^13^C_2_D_3_-Ac) peptides matching TAIR10-annotated protein starts (position 1 or 2) in function of the identity of the ultimate N-terminal residue. **(B)** Distribution of aTIS (*N* = 81) matching the by N-terminal proteomics-identified alternative N-termini with respect to their location to TAIR10 gene models, being either intergenic, located in the 5′ UTR or downstream (dTIS) and in-frame in the CDS. **(C)** Peptide-to-spectrum matches (PSMs) are indicated for the 68 NME-compliant N-termini identified matching in-frame called dTIS of TAIR10 CDSs (Supplementary Dataset S2). *In vivo* Nt-free N-termini, labeled *in vitro* by ^13^C_2_D_3_-Ac, are colored in black, while *in vivo* NTA N-termini matching NatA, NatB, or NatC/E/F specificities are indicated in orange, blue, and green, respectively.

To gain complementary evidence of protein synthesis supporting Ribo-seq-called TIS, we re-analyzed the positional proteomics of *Arabidopsis* cell suspension culture data previously generated (Willems et al., 2017). More precisely, these data concern Nt proteomics datasets of proteomes digested with either trypsin, chymotrypsin, or the endoproteinases Glu-C and Asp-N shown to increase the overall Nt proteome coverage of *Arabidopsis*. In addition to TAIR10 protein sequences, we supplemented our search database with (non-TAIR10) protein sequences corresponding to all *in silico* translations matching Ribo-seq-called TIS. In total, we identified Nt peptides matching 6,493 unique protein N-termini supported by at least two peptide-to-spectrum matches (PSMs; *q* ≤ 0.01) (Supplementary Dataset S2). Of these, 2,041 mapped N-termini (31.4%) corresponded to TAIR10 dbTIS (protein start position 1 or 2). Except for four protein N-termini (20 PSMs, 0.1%), all identified protein N-termini matching dbTIS were compliant with N-terminal methionine excision (NME) enzymatic specificity (Bienvenut et al., 2012) (Figure 2A, for number of PSMs see Supplementary Figure 2). Moreover, based on the number of (*in vitro*) Nt acetylated (NTA) peptides, NTA patterns corresponded with the known specificities and NTA efficiencies of major NAT (NatA, NatB, and NatC/E/F) enzymatic activities in eukaryotes (Ree et al., 2018) (Figure 2A). Taken together, the identified Nt peptides matching dbTIS abide known co-translational enzymatic hallmarks at protein N-termini, which highlights the high quality of the obtained N-terminal proteomic data.

Next to Nt peptides matching annotated TAIR10 N-termini (dbTIS), complementary Nt peptide was evidenced for 81 Ribo-seq-called aTIS (Supplementary Dataset S2; Figure 2B). The majority of these aTIS (i.e., 72 out of 81 aTIS [88.9%]), corresponded to in-frame dTIS in TAIR10 CDS, thus pointing to Nt-truncated proteoforms (Figure 2B). In addition, four identified N-termini matched an aTIS residing in the 5′ UTR reflecting uORF or 5′ leader translation. Lastly, five N-termini matched TIS of intergenic ORFs within the so-called pseudogenes described previously (Willems et al., 2017). Hence, in addition to Ribo-seq evidence, Nt proteomics indicates the common translation of Nt-truncated proteoforms initiated from dTIS within TAIR10 CDSs. Of the 72 N-termini identified pointing to dTIS, four N-termini were, however, identified (16 PSMs, 2.9%) that did not comply with the enzymatic rules of NME, as they exposed large, bulky residues (Ile, Lys, Glu, or Gln). In addition, these four N-termini were initiated from near-cognate start codons and, hence, are more likely to represent possible neo-N-termini and, thus, false positive TIS matches and were not considered further. All the 68 other N-termini identified matching Ribo-seq-called dTIS (529 PSMs, 97.1%) were NME-compliant and showed NTA patterns consistent with the NAT enzymatic rules (Figure 2C). Similar proportions of (modified) N-termini were observed, except for relatively higher proportions of Ac-Thr (7.7% dTIS vs. 2.1% dbTIS) and unmodified Ser N-termini (2.6% dTIS vs. 0.2% dbTIS) (χ^2^ test adjusted *p* < 0.05). Taken together, these Nt peptide identifications and their modification status provide unambiguous complementary proteomics and Ribo-seq proof of the translation of Nt-truncated proteoforms from dTIS for the 68 genes (Table 1). Next to two AGG, two CTG, and a single ATA, ACG, and TTG near-cognate dTIS, AUG was expectedly the most frequent start codon (61/68) among the dTIS (Supplementary Dataset S2). Next to the matching Nt proteomics *Arabidopsis* cell culture data, we cross-referenced publicly available Nt peptide data of *Arabidopsis* stored in the Plant PTM Viewer (Willems et al., 2019) and NTerDB (https://n-terdb.i2bc.paris-saclay.fr) with the Ribo-seq-called dTIS identified in this study. Overall, Nt peptide evidence was also found for 42 out of 68 dTIS-indicative N-termini in various *Arabidopsis* studies, and 34 of these were identified as NTA (Supplementary Dataset S2). An additional 20 Nt peptides identified in various plant tissues matched the Ribo-seq-called dTIS not identified in our Nt COFRADIC analyses (Supplementary Dataset S3). Moreover, all of them were initiated from AUG start codons, and all abided to the NME and NTA enzymatic rules, with 17/20 being NTA except for a Val-starting N-terminus known to be frequently unmodified or partially NTA, and an MP- and MK-starting N-terminus representative of a substrate refractory toward NTA (Goetze et al., 2009) and a typical partial NTA NatC/NatF substrate (Figure 2A). Hence, taking additional advantage of publicly available Nt proteomics data, we were able to obtain univocal riboproteogenomics proof of 88 dTIS-initiated Nt truncated proteoforms, many of which were expressed in a variety of *Arabidopsis* tissues.

**Table 1:** List of 68 N-terminal (Nt)-truncated proteoforms with matching riboproteogenomics evidence shaped by downstream alternative translation initiation sites (dTISs).

**Gene locus**	**Description**	**dTISposition**	**N-terminus**
**tRNA metabolism**			
AT1G14610	Val-tRNA synthetase (TWN2)	Met45	Ac-S
AT1G29880	Gly-tRNA synthetase	Met40	Ac-MD
AT3G11710	Lys-tRNA synthetase (ATKRS-1)	Met17	Ac-MD
AT5G26830	Threonyl-tRNA synthetase (THRRS)	Met34	Ac-A
AT1G52160	tRNAse Z3 (TRZ3)	Met52	Ac-ME
AT2G45330	2′ tRNA phosphotransferase	Met33	Ac-MD
AT1G06560	tRNA methyltransferase 4F (TRM4F)	Met37	Ac-ME
AT1G36310	tRNA methyltransferase 9 (TRM9)	Met29	NH_2_-MR
**Transcription factor, DNA-binding**			
AT1G33060	NAC 014 (NAC014)	Met12	Ac-T
AT1G49480	Related to vernalization1 1 (RTV1)	Met3	Ac-MD
AT1G72210	Basic helix-loop-helix (bHLH96)	Met18	Ac-ME
AT4G22745	Methyl-CPG-binding domain 1 (MBD1)	Met7	NH_2_-MN
AT5G67220	BIM1	Met23	NH_2_-T
**Translation**			
AT1G03360	Ribosomal RNA processing 4 (RRP4)	Met3	NH_2_-MR
AT1G07770; AT3G46040	Ribosomal protein S15A (RPS15A)	Thr105(ACG)->Met	NH_2_-T
AT1G18540; AT1G74050; AT1G74060	Ribosomal protein L6 family protein	Arg25(AGG)->Met	NH_2_-S
AT1G54270; AT3G13920	EIF4A-2	Arg52(AGG)->Met	NH_2_-G
**Metabolism**			
AT1G71180	Probable 3-hydroxyisobutyrate dehydrogenase	Met22	Ac-ME
AT3G44310	Nitrilase 1 (NIT1)	Met7	Ac-S
AT1G58280	Phosphoglycerate mutase family protein	Met43	Ac-ME
AT3G60440	Phosphoglycerate mutase family protein	Met24	Ac-ME
AT5G16440	Isopentenyl diphosphate isomerase 1 (IPP1)	Met59	Ac-T
AT3G02780	Isopentenyl diphosphate isomerase 2 (IPP2)	Met52	Ac-T
AT4G37000	Accelerated cell death (ACD2)	Met41	Ac-ME
AT5G19150	NAD(P)HX dehydratase	Met45	Ac-S
AT5G24400	6-Phosphogluconolactonase 3 (PGL3)	Met70	Ac-A
AT5G36700	2-Phosphoglycolate phosphatase 1 (PGLP1)	Met54	Ac-T
AT3G56490	HIS triad family protein 3 (HIT3)	Met19	Ac-A
AT5G63890	Histidinol dehydrogenase (HDH)	Met18	NH_2_/Ac-MK
AT1G77670	Pyridoxal phosphate-dependent transferase	Met41	Ac-T
AT5G13050	5-Formyltetrahydrofolate cycloligase (5-FCL)	Met43	NH_2_/Ac-S
AT5G12040	ω-amidase	Met63	Ac-A
AT4G08790	Deaminated glutathione amidase	Met29	Ac-A
AT5G03370	Acylphosphatase family	Met66	NH_2_/Ac-T
AT5G15870	Glycosyl hydrolase family 81 protein	Met45	Ac-S
AT5G41970	Metal-dependent protein hydrolase	Met28	NH_2_-A
AT3G10620	Nudix hydrolase homolog 26 (NUDX26)	Met56	Ac-ME
**Phosphorylation**			
AT1G43900	Protein phosphatase 2C family protein	Leu61(CTG)->Met	NH_2_/Ac-T
AT2G23070	Casein kinase II subunit alpha-4 (CKA4)	Leu85(CTG)->Met	Ac-A
AT4G08500	MAPK/ERK kinasekinase 1 (MEKK1)	Met8	Ac-MK
AT3G12200	NIMA-related kinase 7 (Nek7)	Met3	Ac-ME
AT5G11860	SCP1-like small phosphatase 5 (SSP5)	Met45	NH_2_/Ac-MK
**Degradation, proteolysis, and ubiquitination**			
AT2G30110	Ubiquitin-activating enzyme 1 (UBA1)	Met63	NH_2_/Ac-A
AT2G36170	Ubiquitin-60S ribosomal protein L40-1 (RPL40A)	Met84	NH_2_-ML
AT5G46210	Cullin4 (CUL4)	Met26	NH_2_/Ac-MK
AT2G45170	Autophagy 8E (ATG8E)	Met9	Ac-MD
AT4G30920	Leucine aminopeptidase 2 (LAP2)	Met57	NH_2_/Ac-A
AT1G76140	Prolyl endopeptidase	Met65	Ac-G
**Reduction-oxidation processes**			
AT1G60950	Ferredoxin-2 (FD2)	Met52	NH_2_-A
AT2G17420	NADPH-dependent TRX reductase A (NTRA)	Met49	Ac-ME
AT4G19880	Glutathione S-transferase family protein	Met32	NH_2_/Ac-A
AT5G27380	Glutathione synthetase 2 (GSH2)	Met62	Ac-ME
AT2G47730	Glutathione S-transferase phi 8 (GSTF8)	Met49	Ac-A
AT4G11600	Glutathione peroxidase 6 (GPX6)	Met64	Ac-A
AT1G66240	Homolog of anti-oxidant 1 (ATX1)	Met31	Ac-S
AT1G55805	BolA-like family protein	Met52	Ac-S
**Nuclear processes (DNA repair, histone modifications, ..)**			
AT3G23100	Homolog of X-ray repair cross complementing 4(XRCC4)	Met17	NH_2_/Ac-V
AT2G19640	ASH1-related protein 2 (ASHR2)	Ile2(ATA)->Met	Ac-MN
AT5G61140	U5 small nuclear ribonucleoprotein helicase	Met72	NH_2_/Ac-ML
**Others**			
AT5G66675	Protein of unknown function (DUF677)	Met5	Ac-MF
AT5G14540	FLOE1	Met18	Ac-MD
AT3G27310	Plant UBX domain-containing protein 1 (PUX1)	Met22	Ac-ME
AT1G71840	WD-40 repeat family protein	Met10	Ac-MN
AT4G13940	Adenosylhomocysteinase 1 (SAHH)	Leu57(TTG)->Met	NH_2_-S
AT2G39080	NAD(P)-binding Rossmann-fold superfamily protein	Met59	Ac-A
AT2G43290	Calmodulin-like 5 (CML5)	Met47	Ac-ML
AT3G47590	Alpha/beta-Hydrolases superfamily protein	Met50	Ac-MD
AT1G53280	Protein DJ-1 homolog B (DJ1B)	Met48	Ac-S

*For each gene locus, a description and the respective dTIS position (in the main protein isoform) were given, and in the case of translation at near-cognate start codons, the codon/position was provided. The identified NTA (Ac-) and/or Nt-free (NH_2_-) N-terminus was displayed with the two ultimate Nt residues. Genes were assigned to categories based on their function. For more detailed information, (see Supplementary Dataset 2)*.

### *In vitro* Translation Confirms the Expression of Riboproteogenomics-Discovered Nt-Truncated Proteoforms by Alternative Translation Initiation

**Figure 3:**
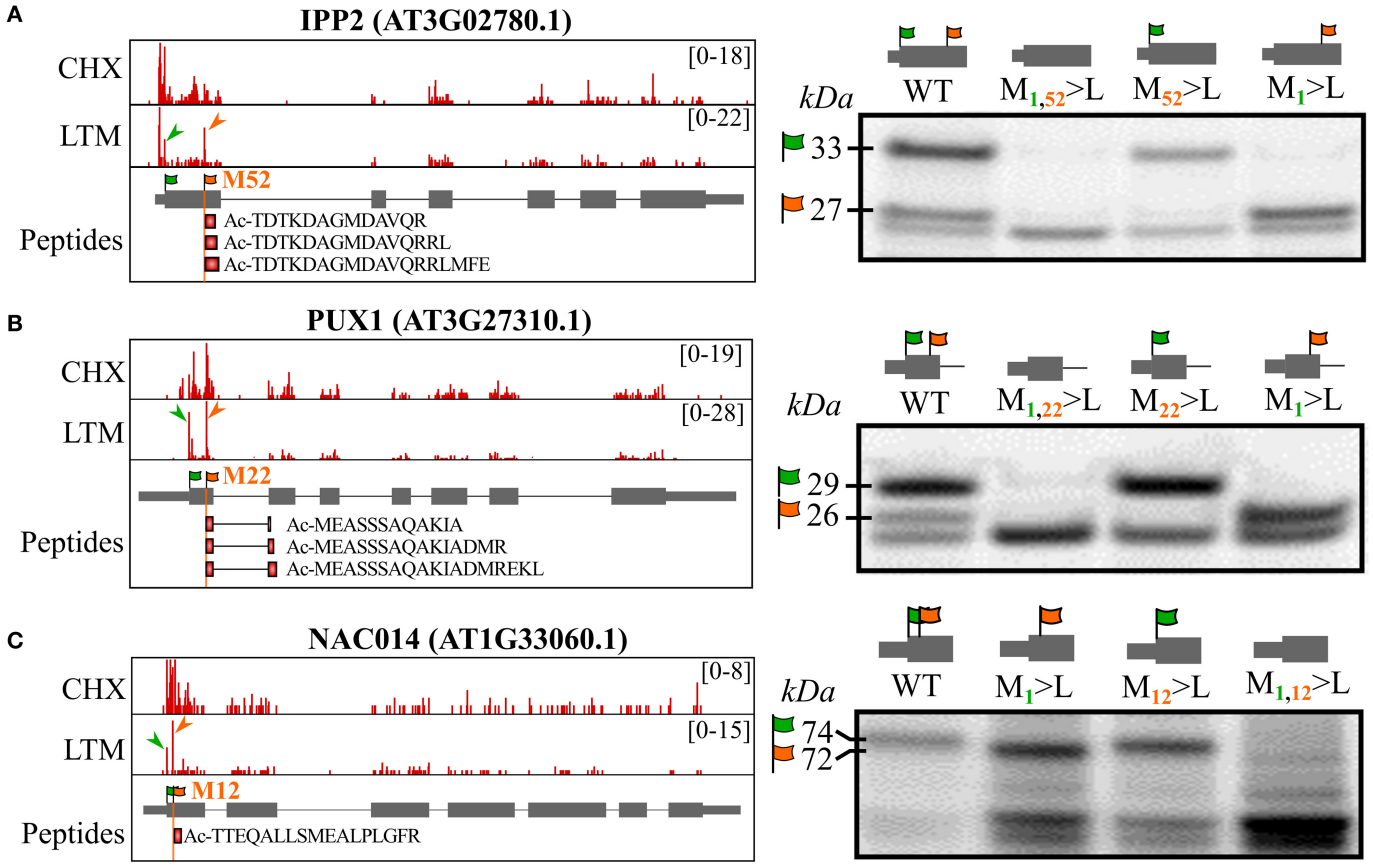
*In vitro* coupled transcription and translation (TnT) of TIS-mutagenized expression constructs confirm translation of the by riboproteogenomics-identified Nt proteoforms. Control and TIS-mutagenized pUNI51 constructs encoding the identified Nt proteoforms of **(A)** *ISOPENTENYL DIPHOSPHATE ISOMERASE2* (*IPP2*, AT3G02780), **(B)** plant *UBX DOMAIN-CONTAINING PROTEIN1* (*PUX1*, AT3G27310), and **(C)** *NAC DOMAIN-CONTAINING PROTEIN14* (*NAC014*, AT1G33060). (Left) Genome views showing CHX and LTM strand-specific positional Ribo-seq data (red). LTM peaks corresponding to Ribo-seq-called dbTIS and dTIS were indicated by green and orange arrowheads, respectively. The vertical orange line indicates the position of the riboproteogenomic-matched dTIS. Nt peptides matching the aTIS event were plotted as red rectangles. In the case of *PUX1*, the Nt peptides span an exon-exon junction (right) (TIS mutagenized) Nt proteoform-encoding constructs were *in vitro* transcribed and translated. Following sodium dodecyl sulfate polyacrylamide gel electrophoresis (SDS-PAGE) and electroblotting, radiolabeled proteins were visualized by radiography. Assignments of the translation products corresponding to translation initiation at the TAIR10-annotated TIS (green flag, M1) and from the identified dTIS (orange flag) were verified by mutating their respective ATG start codons to the (near-cognate start) Leu-encoding codon TTG. In each case, theoretical molecular weights of the identified Nt proteoforms are indicated.

To further support alternative translation initiation at in-frame dTIS as a source for the generation of N-terminal proteoform(s) (pairs), we tested if the mutagenesis of annotated TAIR10 TIS (dbTIS) and/or Ribo-seq-called dTIS would result in altered proteoform expression by *in vitro* coupled transcription and translation (TnT). First, we tested *ISOPENTENYL DIPHOSPHATE ISOMERASE2* (*IPP2*), for which Ribo-seq LTM evidence suggests translation initiation from both the annotated dbTIS and dTIS possibly resulting in the expression of an IPP2 proteoform pair (Figure 3A, green and orange arrowheads, respectively). Alternative translation initiation from a downstream AUG start codon (Met53) indeed resulted in the translation of a 52 AA Nt-truncated proteoform besides the translation of the full-length annotated protein (Figure 3A). In addition, protein synthesis from the identified dTIS was evidenced by three unique NTA peptides (18 PSMs), “TDTKDAGMDAVQR” (trypsin-digested), “TDTKDAGMDAVQRRL” (chymotrypsin-digested), and “TDTKDAGMDAVQRRLFE” (Glu-C-digested), and tryptic Nt peptide evidence previously reported in four *Arabidopsis* studies (Venne et al., 2015; Zhang et al., 2015, 2018; Mielke et al., 2021). Mutation of the dTIS corresponding to the N-terminus starting at AA position 52 and the dbTIS of *IPP2* (resulting in M1 or M52>L mutation) resulted in the exclusive expression of the Nt proteoform translated from the non-mutated TIS (Figure 3A). Second, TnT analysis of the *UBX DOMAIN-CONTAINING PROTEIN1* (*PUX1*) was performed, for which a dTIS matching AA position 22 was identified in our riboproteogenomics analysis (Figure 3B). Also here, three unique NTA peptides matching the dTIS were identified (19 PSMs), further supported by Nt peptide evidence obtained in three other *Arabidopsis* studies (Bienvenut et al., 2012; Zhang et al., 2015, 2018). Furthermore, mutation of the M1 or M22 encoding codons led to exclusive Nt proteoform expression initiated at the dTIS (26 kDa) or dbTIS (29 kDa), respectively. Lastly, we mutated an identified dTIS matching AA position 12 in the transcription factor *NAC DOMAIN-CONTAINING PROTEIN14* (*NAC014)* (Figure 3C) supported by a single NTA peptide (2 PSMs). Next to observing Nt proteoform expression starting at M1 and M13 (74 and 72 kDa, respectively), multiple lower weight proteoforms from possible cryptic downstream TIS increased in abundance upon mutation of TIS-encoding M1 and M13. Taken together, the co-expression of at least two Nt proteoforms (proteoform pair) could be observed for *IPP2, PUX1*, and *NAC014 in vivo* (Ribo-seq and Nt peptide evidence) and *in vitro* (TnT). Besides, mutations of TIS further confirm downstream translation initiation, likely *via* leaky ribosome scanning, giving rise to the expression of Nt-truncated proteoforms and, thus, the possible (co-)expression of proteoform pairs from a single CDS.

### N-Terminal Acetylation Patterns of Chloroplast N-Terminal Proteoforms Differ From Protein N-Termini Raised by Translation Initiation

**Figure 4:**
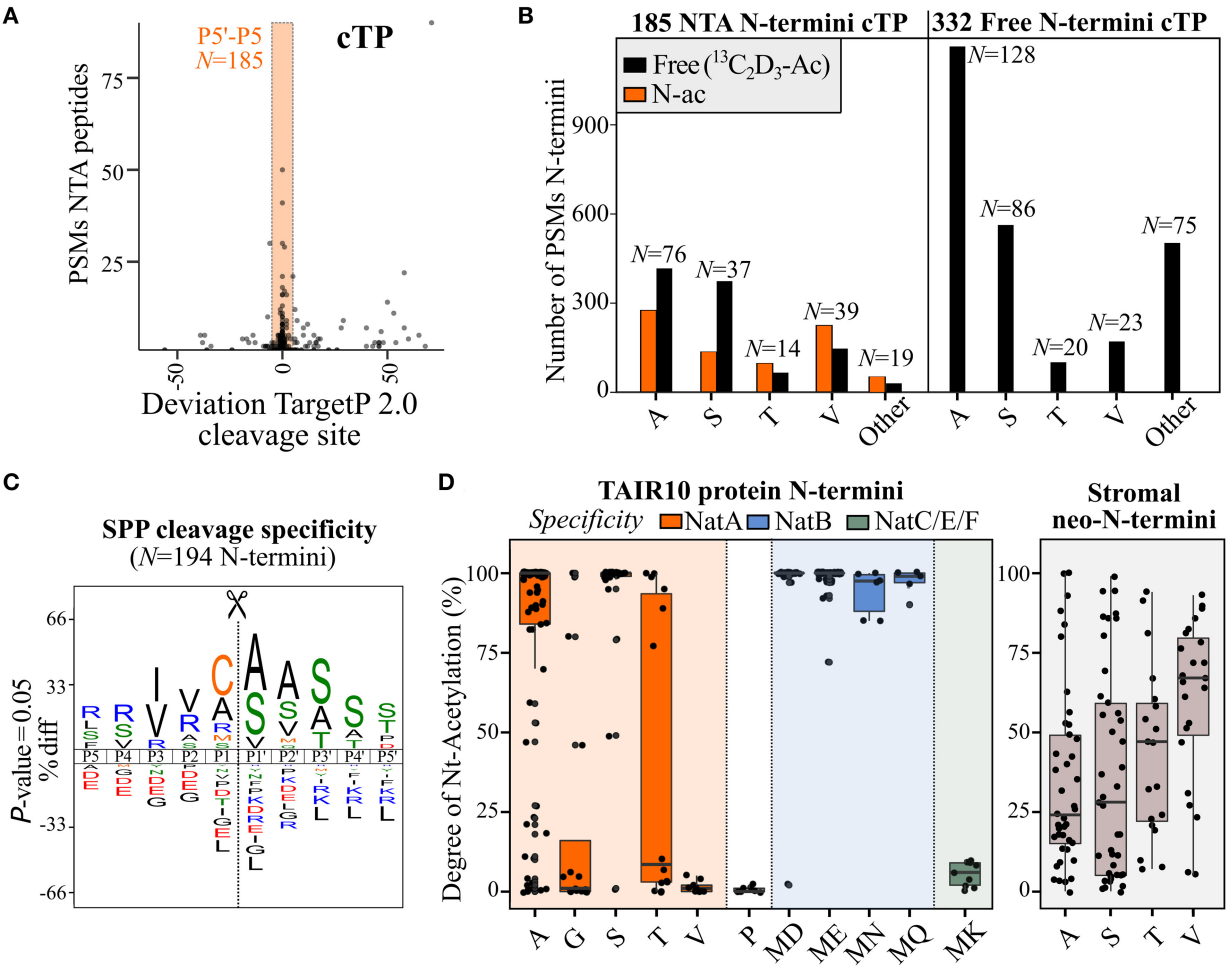
Differences in N-terminal acetylation patterns of N-terminal proteoforms arising from stromal processing peptidase (SPP) processing or (alternative) translation initiation. **(A)** PSMs of NTA peptides identified in cell cultures in function of the deviation to the chloroplast transit peptide (cTP) P1′ cleavage site predicted by TargetP 2.0 (Almagro Armenteros et al., 2019). The orange rectangle reflects the P5–P5′ predicted region, considered in this study to assign neo-N-termini indicative of cTP cleavage. **(B)** Number of identified PSMs of identified NTA [and corresponding Nt-free (^13^C_2_D_3_-Ac), if any] Nt peptides matching considered cTP cleavages [see panel **(A)**] in the case of N-termini exclusively identified as Nt-free (right) or identified by at least one PSM as NTA (left). Only Ala, Ser, Thr, and Val neo-N-termini (number indicated above bar) were plotted, as these represent the majority of cTP cleavage neo-N-termini (3,739/4,322 PSMs, 87%). **(C)** iceLogo (Colaert et al., 2009) motifs of cleavage motifs (P5–P5′) with an Nt peptide start position deviating maximally five residues of the predicted chloroplast or mitochondrial transit peptide (cTP/mTP) cleavage site (Almagro Armenteros et al., 2019) and with a SUBA4 chloroplast consensus location (Hooper et al., 2017). In case multiple cleavage sites were identified within a 5-residue window, the most upstream neo-N-terminus was selected as representative member. **(D)** Degree of NTA (%) for annotated protein N-termini (left) and stromal neo-N-termini (right) was plotted for various types of N-termini according to their NAT specificity profiles. N-termini matching NatA, NatB, or NatC/E/F specificities are indicated in orange, blue, and green, respectively, while stromal protein NTA is indicated in gray. Only Nt residue(s) with at least five data points were plotted; for full overview (see Supplementary Dataset S4).

Of the 2,135 identified NTA N-termini (at least one PSM), 426 (20%) N-termini were not directly indicative of translation initiation, as they did not match dbTIS or Ribo-seq-called dTIS (Supplementary Dataset 2). Plotting the number of PSMs of identified NTA N-termini according to their start position in the protein sequence clearly shows, besides dbTIS density (protein position 1/2), increased density in the Nt protein region (e.g., position < 100) (Supplementary Figure 3A). Exceptionally, in photosynthetic species, after processing of their sorting signals, chloroplastic localized proteins can be post-translationally NTA by GCN5-related *N*-acetyltransferase (GNAT) domain-containing proteins, which show diverse NAT substrate specificities (Rowland et al., 2015; Bienvenut et al., 2020). In accordance, the protein position of 185 of the 426 non-TIS-called NTA N-termini (43%) deviated <5 AA residues from the TargetP 2.0 (Almagro Armenteros et al., 2019). SPP predicted cleavage site corresponding to the removal of the cTP (Figure 4A). However, 149 out of 185 of these NTA peptides also had corresponding Nt-free peptide identifications, which, in the case of Ala and Ser N-termini, were more abundant when considering PSMs opposed to Val and Thr N-termini (Figure 4B, left). Furthermore, 332 additional N-termini matching SPP cleavages (and with 513 N-termini matching cTP cleavages identified in total) solely were identified as Nt-free (Supplementary Dataset S2; Figure 4B, right). Aside from predicted cTP, the start position of another 25 NTA N-termini deviated <5 AA residues from predicted mitochondrial transit peptide (mTP) cleavage sites, while none matched signal peptide and luminal transit peptide predicted cleavages (Supplementary Figure 3B). As no NTA enzymatic activity is currently known in mitochondria (Giglione and Meinnel, 2021), these may hint at ambiguous targeting peptides representing cTP instead of mTP sequences or, alternatively, point to dual protein localization explained by the expression of distinct Nt-modified proteoforms (e.g., the NTA and Nt-free proteoform variants localizing in the chloroplasts and mitochondria, respectively).

To relate our *Arabidopsis* cell culture findings with *in vivo* degrees of NTA and to confirm the location of NTA neo-N-termini indicative of post-translational cTP-processing in the chloroplast, we additionally performed Nt COFRADIC analyses on proteomes of enriched chloroplasts and corresponding stromal protein fractions from *Arabidopsis* leaves. Both fractions were processed in two different ways. First, as performed for cell cultures (Willems et al., 2017), heavy ^13^C_2_D_3_-acetate labeling was performed *in vitro*, which enables quantitative determination of the degree of *in vivo* NTA, equaling the ratio of light over heavy NTA peptide precursor intensity (Δ 5 Da) (Van Damme et al., 2011, 2013). Second, another Nt COFRADIC analysis was performed where the *invitro*^13^C_2_D_3_-acetate labeling step was omitted. This leaves non-NTA peptide N-termini unmodified, thereby solely enriching *in vivo* NTA N-termini. In total, 311 natural NTA peptides were identified in the chloroplast and 848 in stroma, the latter enriched for natural NTA peptides by serving a sink of cTP-processed NTA neo-N-termini, with 127 NTA peptides common to both (Supplementary Dataset S4). A total of 297 (35%) NTA peptides correspond to cTP cleavage processing events (within the P5–P5′ region of predicted cTP cleavage sites). Of these, 103 NTA peptides were previously identified in *Arabidopsis* cell cultures and, thus, corroborate post-translational NTA in the chloroplast as additional NTA source in the riboproteogenomics analysis. In addition, 26 NTA peptides matched mTP cleavage sites, of which the corresponding Nt proteoforms are likely dual localized or incorrectly predicted by TargetP 2.0 as indicated before. As trimming of neo-N-termini by aminopeptidases is prevalent in the chloroplast (Rowland et al., 2015), we used the likely primary SPP cleavage site by selecting the most upstream NTA neo-N-terminus with a start position in the P5–P5′ region of predicted cTP/mTP cleavages of proteins assigned a chloroplastic consensus localization according to the subcellular localization database for *Arabidopsis* proteins SUBA4 (Hooper et al., 2017). This resulted in 194 NTA representative neo-N-termini, with frequent occurrences of Cys, Ala, Arg, and Met in the P1 position and Ala, Ser, and Val in P1′ (Figure 4C). While Cys, Ala, and Met were observed earlier at P1 of neo-N-termini generated upon cTP cleavage in *Arabidopsis*, Arg and other basic residue occupancies were suggested earlier by considering a high-confident subset of recombinant primary SPP cleavage sites (Rowland et al., 2015). In addition, enrichment of Ala and Cys at P1, as well as Val and Ile at P3, matches the characteristic SPP P3–P1′ cleavage motif reported before, (V/I)-X-(A/C)↓A (Gavel and Von Heijne, 1990). Despite SPP cleavage site hallmarks, it is, however, likely that aminopeptidase activity still complicates delineation of primary SPP cleavage motifs (Figure 4C).

Next to SPP cleavage events, and while rationally fewer than in global N-terminome analyses, 503 NTA peptides matching annotated protein N-termini (position 1 or 2) were also identified. Besides the 17 annotated protein N-termini of chloroplast-encoded genes, the remainder are from cytosolic origin and were likely identified as impurities in the chloroplast isolation procedure as also evidenced by their general greater cellular abundance. For all NTA peptides, the degrees of NTA were calculated (Supplementary Dataset S4) and plotted for dbTIS-indicative protein N-termini (position 1 or 2). The degree of NTA largely agrees with the earlier observed PSM counts of protein N-termini in cell cultures (Figure 2A) and known enzymatic NAT efficiencies (Figure 4D, left), with high degrees of NTA of Ser and Ala N-termini (average 94.6% and 80.3%, respectively) [NatA substrates (Linster et al., 2015)] and MD/ME/MN/MQ N-termini (NatB) (Van Damme et al., 2012). For Thr N-termini, there seems to be a bimodal distribution of high and low NTA N-termini, while the degree of NTA is generally low for Gly (21.4% NTA on average), Val (1.4%) (NatA) and MK (5.4%) N-termini (NatF) (Van Damme et al., 2011), and, as expected, Pro N-termini were essentially Nt-free (Goetze et al., 2009). Next, we inspected the degree of post-translational NTA of all unique stromal neo-N-termini. Sufficient NTA peptide identifications were evident for Ala, Ser, Val, and Thr neo-N-termini, clearly showing a differing degree of NTA compared to annotated protein N-termini. While the NTA degree was generally lower in case of Ser and Ala (36.3 and 34.9%, respectively), it was higher for Val (60.3%) (Figure 4D, right), and the distribution of Thr was rather centered as compared to the bimodal distribution observed for annotated protein N-termini. Similar co- and post-translational NTA degrees of (neo-)N-termini were evident in both the chloroplast fractions analyzed, albeit with less observations in case of whole chloroplasts (Supplementary Figure 4). In addition, this quantitative NTA analysis is in line with observed NTA and Nt-free PSM identifications in cell cultures (Figure 4B, left). The deviating NTA patterns of stromal neo-N-termini vs. translation-indicative Ala, Ser, Thr, and Val N-termini likely reflect differing enzymatic specificities and efficiencies of the responsible chloroplast GNAT as compared to cytosolic NatA; the latter NAT is responsible for the co-translational NTA of Ala, Ser, Thr, and Val N-termini. Taking together, post-translational NTA in the chloroplast forms a major source of NTA peptides in plants and shows different enzymatic preferences, and thus NTA patterns, compared to co-translational NTA. These results are in line with previous reports (Rowland et al., 2015; Bienvenut et al., 2020).

### Alternative Translation Initiation Governing Dual Localization of Cytosolic and Organellar N-Terminal Proteoforms

**Figure 5:**
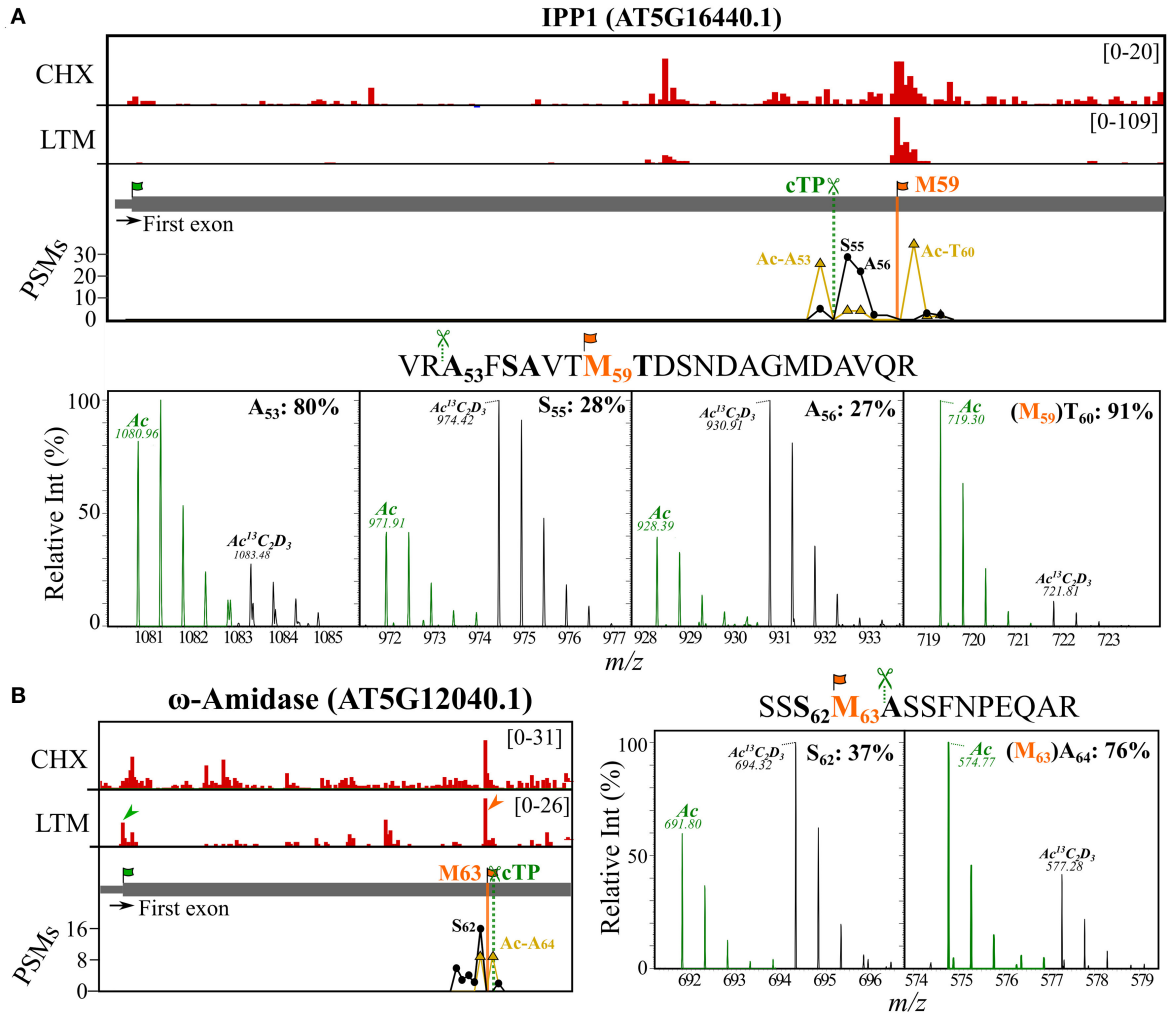
Riboproteogenomic evidence indicative of proteolytic signal peptide processing and alternative TIS usage. Ribo-seq coverage and PSM counts of identified Nt peptides were plotted for the first exonic region of [**(A)**, top] *IPP1* (AT5G16440) and [**(B)**, left] ω-amidase (AT5G12040). Genome view showing CHX and LTM strand-specific positional Ribo-seq data (red). Vertical lines indicate identified dTIS (orange) and the cTP predicted cleavage site (dark green, dotted line) (Almagro Armenteros et al., 2019). LTM peaks corresponding to Ribo-seq-called dbTIS and dTIS were indicated by green and orange arrowheads, respectively. The PSM counts for the *in vivo* NTA (yellow) and *invitro*^13^C_2_D_3_-NTA (i.e., *in vivo* Nt-free) (black) N-termini are plotted. MS1 spectra of uniquely identified NTA peptides originating from [**(A)**, bottom] *IPP1* (AT5G16440.1) and [**(B)**, right] ω-amidase (AT5G12040.1). Isotopic envelopes matching the *in vivo* NTA (green) and *in vitro* Ac^13^C_2_D3 (black, +5 Da) counterparts are shown for different NTA Nt peptides identified (peptide on top, bold residues). The calculated degree of NTA (%) was indicated together with the corresponding Nt residue. Predicted cTP cleavage motifs (P2–P1) preceding the identified Nt peptide sequences as well as the initiator Met (iMet) of riboproteogenomic-mapped dTIS (orange) were indicated in the sequence. All peptide precursors had a 2+ charge, and precursor *m/z* values are indicated.

Thus far, we have shown that NTA peptides matching internal protein start positions may either result from protein synthesis at dTIS (because of co-translational NTA) (Figure 2C) or, alternatively, represent mature neo-N-termini of nuclear-encoded chloroplastic proteins that were post-translational NTA (Figures 4B,D). However, in some cases, it is complicated to attribute NTA peptides to either process. For instance, Met residues are well-represented in the P1 position of stromal NTA neo-N-termini (30/150 sites [20%]), therefore, representing possible TIS (Figure 4C) and 34 out of 68 (50%) proteins with a dTIS called and matching Nt peptide evidence had predicted TargetP 2.0 cleavage sites (17 cTP, 16 mTP, and single luTP). This is significantly higher as expected, since cTP/mTP/luTP are predicted for approximately 10% of the TAIR10 proteome by TargetP 2.0. Moreover, for the majority of these proteins (26 or 76%), the predicted TargetP 2.0 cleavage site deviated <10 residues from the corresponding dTIS called (Supplementary Dataset S2). For instance, the Ribo-seq-called dTIS of *IPP2* (Figure 3A) exactly matched a predicted cTP cleavage with M52 at P1. Hence, NTA either occurred co- or posttranslational in the cytosol or chloroplast stroma, respectively, or both scenarios could have occurred. In line, for its close homolog *IPP1*, NTA peptides were identified in cell cultures starting at Ala60 and Ser53, corresponding respectively to a Ribo-seq-called dTIS at Met59 and a predicted cTP cleavage site at position 54 (Figure 5A, top). Moreover, (neo-)N-termini matching the by Ribo-seq-identified dTIS (Thr60 starting N-terminus) or cTP cleavages (Ala53, Ser55, and Ala56 starting N-termini) were found in the chloroplast leaf extracts. In chloroplast stroma, an NTA degree of 80% was identified for Ala53, whereas lower percentages of NTA were observed for Ser55 (28%) and Ala56 (27%) (Figure 5A, bottom; Supplementary Dataset S4). Hence, the presumably primary SPP-exposed Ala53 neo-N-terminus shows a higher NTA degree compared to its downstream, aminopeptidase-processed proteoforms, possibly due to the kinetics of ensuing Nt modifications. In addition, the highest degree of NTA was observed in the case of the Thr60 (91%) Nt peptide raised by alternative translation initiation at the dTIS corresponding to Met59 and subsequent co-translational removal of this iMet by the action of MetAPs. Confirming our results, for both *IPP* homologous genes, a long and short transcript has previously been discovered in *Arabidopsis* because of alternative transcription start sites, encoding full-length proteins targeted to mitochondria or chloroplasts, or Nt truncated cytosolic proteoforms (Phillips et al., 2008). In addition, Nt peptides supporting dTIS as well as organellar import processing were also found for ω-amidase (AT5G12040.1). In fact, an alternative start codon corresponding to Met63 was suggested earlier as a possible dTIS (Zhang and Marsolais, 2014) and here verified in cell cultures by Ribo-seq and NTA peptide evidence matching the iMet-processed N-terminus at Ala64 (Figure 5B, left). In addition, Ala64 was predicted as a cTP cleavage site and was found to be partial NTA (76%) in stromal fractions (Figure 5B, right). In addition, we identified an NTA peptide upstream at Ser61 in the stromal fractions, likely representing the primary exposed SPP cleavage site that was NTA to a lower extent (37%). This NTA percentage is in line with the ratio of Nt-free (16) over NTA PSMs (9) for the neo-N-terminus identified in cell cultures (Figure 5B, left). Taken together, downstream in-frame translation start sites are frequently found for proteins targeted to the chloroplast and/or mitochondrion and dTIS thereby function as a (regulatory) mechanism for acquiring functional protein copies in the cytosol (i.e., translated proteoforms lacking signal sequences of their full-length counterparts) as well as in organelles (i.e., translated proteoforms encompassing signal sequences). Moreover, the degree of NTA can provide an additional cue to distinguish Nt proteoforms originating from alternative translation or organellar processing.

## Discussion

Ribosome sequencing (Ribo-seq) represents a powerful technique to empirically determine translated regions in the genome, and has revealed yet an unexplored proteome complexity. Using inhibitors that halt ribosomes during translation initiation, TIS can be uncovered on a genome-wide scale (Ingolia et al., 2011; Lee et al., 2012). Obtained Ribo-seq coverage can be exploited by machine learning algorithms for delineating ORFs (Crappe et al., 2015; Zhang et al., 2017; Verbruggen et al., 2019). Subsequently, translation products from the ORFs proposed by Ribo-seq can be matched by proteomics data, designated here as riboproteogenomics, further providing unequivocal proof of protein synthesis. Furthermore, positional proteomic approaches that enrich for Nt peptides are especially complementary, as they can confirm TIS mapped by Ribo-seq, thereby serving as proxies of translation initiation. Such confirmation is of prime importance in proteogenomics and gene annotation, where control for false positive identifications is a major issue (Nesvizhskii, 2014). In our previous survey, we have utilized a riboproteogenomics approach for identifying intergenic protein start sites in the *Arabidopsis* genome (Willems et al., 2017). In this study, we focused on a TIS located internally in the protein CDS and in-frame with annotated TIS, i.e., downstream TIS (dTIS) that can give rise to Nt-truncated proteoforms. To delineate translated ORFs and assign TIS in *Arabidopsis*, we used PROTEOFORMER (Crappe et al., 2015), overall resulting in 29,013 Ribo-seq-called TIS. Of these, 13,324 (45.9%) corresponded to TAIR10/Araport11-annotated TIS. With reference to dbTIS, there were 135 and 52 TIS solely mapping either TAIR10 or Araport11 start codons, respectively (Supplementary Dataset S1), highlighting the inconsistencies related to TIS calling in automated gene annotation algorithms that typically are biased toward AUG start codons, and selection of the longest ORF (Saeys et al., 2007). For instance, it is not excluded that some TIS reported as dTIS in this study might represent unassigned dbTIS. Conversely, annotated TIS might represent dTIS in case of Nt extended proteoforms. Of interest, some genes with alternatively spliced transcripts had multiple annotated TIS supported by Ribo-seq, with 14 genes having two annotated TIS with more than 20 LTM reads (Supplementary Dataset S1). For instance, an uncharacterized transmembrane gene AT2G04360 showed LTM peaks at two TIS matching translation initiation on different transcripts (Supplementary Figure 5). Next to the Ribo-seq-aided identification of translation initiation at dbTIS, a vast, unannotated TIS landscape was discovered in *Arabidopsis* with high proportions (~80%) of non-AUG start codons (Figures 1B,C). This finding is in line with recent Ribo-seq reports on *Solanum lycopersicum* (tomato) that similarly employed LTM for halting initiating ribosomes (Li and Liu, 2020). Such TIS frequently point to uORF translation, as 7,572 called TIS resided in so-called 5′ UTRs, corroborating earlier observations that uORFs are found in 35 to 50% of the *Arabidopsis* genes (Von Arnim et al., 2014; Niu et al., 2020). Occasionally, such events may point to missed exonic annotation in available transcript structures (Willems et al., 2017). The regulatory role of uORF translation and the translation of novel, intergenic ORFs were addressed before in *Arabidopsis* (Liu et al., 2013; Willems et al., 2017) and not further considered here. Instead, we focused on the 7,653 called TIS located downstream and in-frame within the CDS of 2,818 genes (Supplementary Dataset S1), representing possible dTIS that could give rise to Nt-truncated proteoforms. A previous Ribo-seq profiling in *Arabidopsis* seedlings used increased CHX ribosome footprint density typically observed at start codons (also apparent in this study, Figure 1A) to suggest 35 plausible dTIS in 31 genes (Liu et al., 2013). Note that we observed an asymmetric distribution for both CHX and LTM footprints in genes, with decreasing density toward the 3′ end of genes (Figure 1A), which has also been observed (to some extent) in other Ribo-seq profiling studies (Ingolia et al., 2009; Li and Liu, 2020). Noteworthy, of the 35 plausible dTIS suggested in *Arabidopsis* seedlings (by making use of increased CHX ribosome footprint densities at starts) (Liu et al., 2013), 18 dTIS (> 50%) were also called in our study. This included a dTIS corresponding to Met64 of glutathione peroxidase 6 (GPX6) (Supplementary Figure 6A), a protein that was shown before to be dual localized to mitochondria and cytosol, thus further pointing to alternative translation initiation and the expression of multiple Nt proteoforms as the origin of this observed phenomena (Attacha et al., 2017). All considering, the large agreement with earlier reported dTIS (Liu et al., 2013) besides matching Nt peptide evidence is indicative of the validity of our TIS calling strategy.

In the next phase, PROTEOFORMER (Crappe et al., 2015) was used to generate a Ribo-seq-based custom protein database. Here, Ribo-seq alignment settings and TIS calling thresholds were optimized to maximize the peptide identification rate of annotated proteins (see section Materials and Methods). For additional proof of translation initiation at dTIS and resulting Nt proteoform expression, Nt peptide identifications can further corroborate Ribo-seq-mapped TIS. Furthermore, because of co-translational NME and NTA occurring on nascent peptide chains, known enzymatic specificities associated with translation initiation can be used as a biological filter to select Nt peptides indicative of translation initiation (Willems et al., 2017). Next to abiding NME enzymatic rules, the identification of dbTIS-indicative NTA Nt peptides in *Arabidopsis* cell cultures (Figure 2A) and their degree of NTA (Figure 4D) are well in-line with known eukaryotic NAT specificities and activities reported on protein N-termini (Linster et al., 2015). Of the 72 identified N-termini matching identified dTIS events in *Arabidopsis* cell cultures, 68 followed NME and NTA rules (Table 1). Hence, strengthening the confidence that translation initiation at dTIS results in translation of Nt-truncated proteoforms. It should be noted that it in some cases, it is possible that dbTIS were incorrectly annotated, and that the identified “dTIS” actually might represent the correct TIS. For instance, Met17 likely represents the actual start site of the DNA repair protein homolog *XRCC4* (AT3G23100) given the absence of preceding Ribo-seq signal and lack of peptide evidence in the *Arabidopsis* Peptide Atlas (Van Wijk et al., 2021) despite theoretical likely identifiable tryptic peptides (Supplementary Figure 7). In contrast, the majority of genes (62/68) with a dTIS called did have a matching Ribo-seq-called dbTIS (Supplementary Dataset S1), and for seven genes, Nt peptides were identified matching both the dTIS and dbTIS, thus indicative of protein translation of both Nt proteoforms. Cross-referencing public Nt proteomic data stored in the Plant PTM Viewer (Willems et al., 2019) and NTerDB (https://n-terdb.i2bc.paris-saclay.fr/) confirmed translation initiation at 42 dTIS (Supplementary Dataset S2) while providing additional Nt peptide evidence for an additional 20 Ribo-seq-supported dTIS (Supplementary Dataset S3). Note that in our study we only considered dTIS proteoforms supported by proteomics. It stands to reason that dTIS events with solely Ribo-seq evidence inform more generally on true Nt-truncated proteoforms, the majority of which, however, remain non-identified by proteomics because of multiple plausible reasons, such as MS incompatibility (e.g., precursor mass outside detectable *m*/*z* range, suboptimal ionization, low abundance, and/or high hydrophobicity) or downstream processing (e.g., removal of signal peptide sequences).

While complementary Ribo-seq and Nt peptide evidence are clear hallmarks of dTIS events, the functional relevance of Nt proteoforms resulting from downstream translation initiation, and especially the possible expression of Nt proteoform pairs in plants remains largely unexplored. The expression of some of the by riboproteogenomics-discovered proteoform pairs was additionally confirmed by *in vitro* coupled transcription and translation (TnT) (Figure 3). In fact, in case of the identified dTIS (Met45) of an NAD(P)HX dehydratase (AT5G19150), translation of an Nt truncated proteoform was previously confirmed using a similar mutagenesis approach (Niehaus et al., 2014). Furthermore, while full-length NAD(P)HX dehydratase was shown to be targeted to the chloroplast and mitochondria, the Nt-truncated proteoform was localized in the cytosol (Niehaus et al., 2014). Besides, several other of the 68 dTIS (Table 1) were already reported reported or suggested to give rise to Nt-truncated proteoforms and/or dual localization (see Supplementary Table 2). Similar to the Nt proteoform pairs of the *IPP* homologs (Phillips et al., 2008), multiple *GSTF8* and *GSH2* transcription start sites (TSSs) resulted in alternative transcripts, encoding cTP-containing proteoforms targeted to the chloroplast and Nt-truncated cytosolic proteoforms lacking a cTP (Wachter et al., 2005; Thatcher et al., 2007). In addition to alternative TSSs, leaky scanning can give rise to protein synthesis from dTIS. This translational regulation has already been reported to occur in the case of multiple *Arabidopsis* aminoacyl-tRNA synthetases (aaRSs), and to serve as a mechanism for dual protein localization to the mitochondria and cytosol (Garin et al., 2020). In this study, we obtained riboproteogenomics data for three such aaRSs, namely, *ValRS* (AT1G14610), *ThrRS* (AT5G26830), and *GlyRS* (AT1G29880) (Souciet et al., 1999; Duchene et al., 2001). Intriguingly, we also identified dTIS for other tRNA metabolic enzymes (Table 1). For instance, a dTIS (Met52) for the tRNase Z3 (AT1G52160) was identified (Supplementary Figure 6B), an enzyme with a reported dual nuclear and mitochondrial localization (Canino et al., 2009). Similarly, for yet another tRNA-metabolic enzyme, the 2′ tRNA phosphatase (AT2G45330), a dTIS (Met33) bypassed the predicted mTP (Supplementary Figure 6C). As such, tRNA-metabolic genes are likely subject to alternative subcellular localization *via* dTIS, similar to what was reported for aaRSs in *Arabidopsis* (Souciet et al., 1999; Duchene et al., 2001). Interestingly, several identified dTIS mapped to metabolic enzymes, which might, thus, serve as an effective mechanism to partition enzymes between compartments, thereby addressing metabolic needs in different organelles. Lastly, previous reports have shown conservation of dTIS events in multiple species (Bazykin and Kochetov, 2011; Guirimand et al., 2012; Van Damme et al., 2014; Zhang and Marsolais, 2014). For instance, the ω-amidase (AT5G12040.1) initiated at Met63 and identified in this study (Figure 5B) is suspected to match a dTIS governing cytosolic and mitochondrial localization based on its human ortholog Nt proteoforms reported (Zhang and Marsolais, 2014). The evolutionary conservation of dTIS can be intriguing, especially in plants that underwent chloroplast to nucleus gene transfer, as, for instance, the conserved *Arabidopsis IPP* dTIS correspond to the dbTIS of the *IPP* orthologs in the green algae *Chlamydomonas reinhardtii* and lycophyte *Selaginella moellendorfii* (Supplementary Figure 8).

Next to translation initiation-indicative Nt peptides, a large number of Nt peptides reporting on proteolytic cleavages (neo-N-termini) are typically identified in Nt proteomic experiments (Perrar et al., 2019). In this study, when considering all neo-N-terminal *Arabidopsis* peptides identified and a maximal deviation ≤ 5 AA residues of TargetP2.0 predicted cleavages, more than 1,000 unique N-terminal sorting signal processing sites (1,043) were identified (Supplementary Datasets S2, S4). Intriguingly, neo-N-termini exposed by SPP can be post-translational NTA (Zybailov et al., 2008; Bienvenut et al., 2012, 2020; Rowland et al., 2015). Thereby, aside from NTA peptides originating from protein synthesis, in plants, NTA peptides frequently match stromal neo-N-termini (Figure 4A), and of the 1,043 unique N-terminal sorting signal processing sites identified, 437 were identified with at least 1 NTA PSM. To quantitively study the degree of NTA in plant chloroplasts, a quantitative NTA study on chloroplast and protein stromal-enriched fractions was performed to determine the stoichiometry of *in vivo* NTA (%) of annotated protein N-termini and stromal neo-N-termini (Supplementary Dataset S4). This analysis revealed a differing degree of NTA associated with co-translational vs. post-translational NTA accompanying protein synthesis and chloroplast import, respectively. Intriguingly, the extent of NTA of Val neo-N-termini in the stroma drastically exceeds that of native protein N-termini (Figure 4D). This is in line with a previous report (Rowland et al., 2015) and is likely attributed to differing specificity and efficiency of choroplastic vs. cytosolic NATs (Bienvenut et al., 2020). Of interest, recombinant GNAT2 showed the highest preference for Thr and Val residues (Bienvenut et al., 2020), perhaps at least partially accounting for the higher degree of NTA on Val neo-N-termini (Figure 4D). As such, in the case of close proximity of dTIS and cTP cleavage site predictions, NTA features could additionally aid to distinguish between the co- and post-translational origins of NTA peptides, although further experimental evidence would be required to validate their origin. Interestingly, NME-compliant NTA N-termini of two chloroplast-encoded annotated protein N-termini (i.e., Thr2 of PHOTOSYSTEM II REACTION CENTER PROTEIN L (PSBL) and Ala2 of PHOTOSYNTHETIC ELECTRON TRANSFER A (PETA) (Supplementary Dataset S4) were also identified as being partial NTA and further supported by public N-terminomics data for PSBL (Soh et al., 2020). Also noteworthy is that while the NTA patterns of dTIS-indicative N-termini appear largely similar to those of dbTIS-indicative N-termini, for instance, a 100% NTA for the Gly-tRNA synthetase dTIS at Met40 that has an “MD” N-terminus (Supplementary Table 3), for other dTIS-indicative N-termini, a lower degree of NTA might possibly be explained by the gradual loss of associated NATs during leaky ribosome scanning. Furthermore, in the case of ω-amidase (Figure 5B), the predicted cTP cleavage site coincides with the dTIS, and the readout of NTA can, thus, be a read-out of both generated N-termini.

## Data Availability Statement

The datasets presented in this study can be found in online repositories. The names of the repository/repositories and accession number(s) can be found in the article/Supplementary Material.

## Author Contributions

PW and PVD conceived the research. VJ and PVD conducted the experiments. PVD performed the proteomics. PW, EN, and PVD analyzed the data. PW, FV, and PVD wrote the article. All authors contributed to the article and approved the submitted version.

## Funding

This study was supported by the European Research Council (ERC) under the European Union's Horizon 2020 research and innovation program (PROPHECY grant agreement no. 803972 to PVD), the Research Foundation-Flanders (Junior Postdoctoral fellowship grant no. 12T1722N to PW) and the Research Foundation-Flanders-Fonds de la Recherche Scientifique (Excellence of Science project no. 30829584 to FV).

## Conflict of Interest

The authors declare that the research was conducted in the absence of any commercial or financial relationships that could be construed as a potential conflict of interest.

## Publisher's Note

All claims expressed in this article are solely those of the authors and do not necessarily represent those of their affiliated organizations, or those of the publisher, the editors and the reviewers. Any product that may be evaluated in this article, or claim that may be made by its manufacturer, is not guaranteed or endorsed by the publisher.
