# Supplementary material for: To New Beginnings: Riboproteogenomics Discovery of N-Terminal Proteoforms in Arabidopsis Thaliana
Source: Front Plant Sci. 2022 Jan 6;12:778804. doi: 10.3389/fpls.2021.778804 (PMC8770321; doi:10.3389/fpls.2021.778804)
Supplement: Supplementary file 1 [file Data_Sheet_1.zip › Supplementary Figures and Tables.PDF]

Supplementary Material

1 Supplementary Data

The PROTEOFORMER generated MySQL database and its derived protein FASTA database, RAW proteomics data of the NTA degree in plant chloroplasts and stromal fractions, and peptide identification results are available under the Open Science Framework project 'ajx5e' (https://osf.io/ajx5e/).

Supplementary Datasets S1-S4 are added as separate files.

2 Supplementary Figures and Tables

2.1 Supplementary Figures

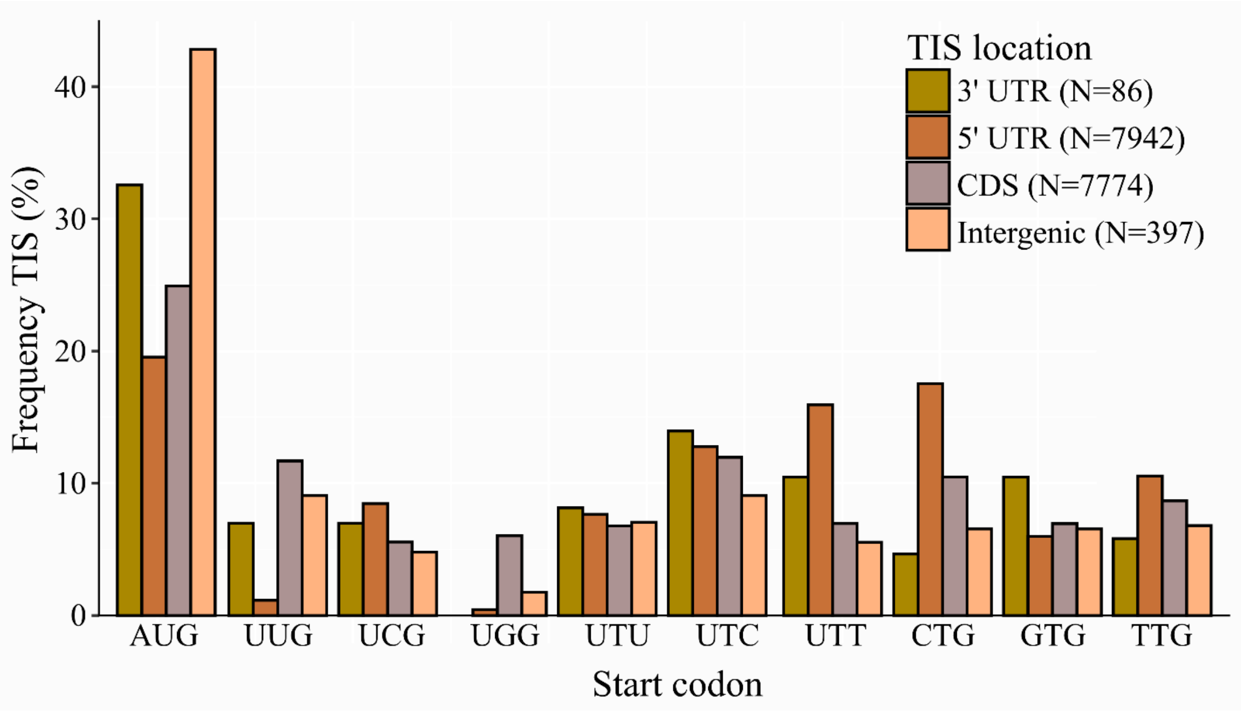

Supplementary Figure 1| Start codon distribution of non-annotated Ribo-seq called TIS according to genomic location (3' UTR, 5' UTR or CDS in annotated gene structures or intergenic regions).

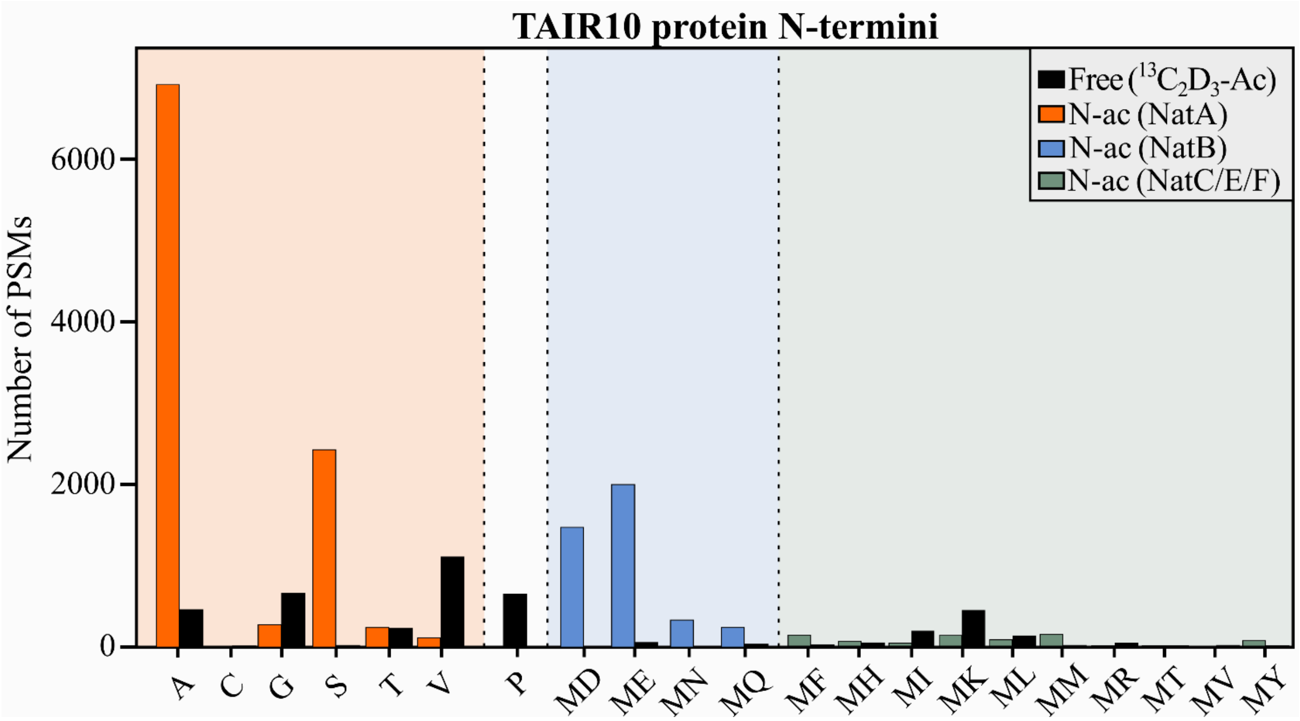

Supplementary Figure 2| Number of PSMs corresponding to identified NTA or Nt-free ($^{13}C_2D_3$-Ac) Nt peptides matching TAIR10 annotated protein starts (position 1 or 2) in function of the identity of the ultimate N-terminal residue. *In vivo* Nt free N-termini – labeled in vitro by $^{13}C_2D_3$-Ac – are colored in black, while in vivo NTA N-termini matching NatA, NatB or NatC/E/F specificities are indicated in orange, blue and green, respectively.

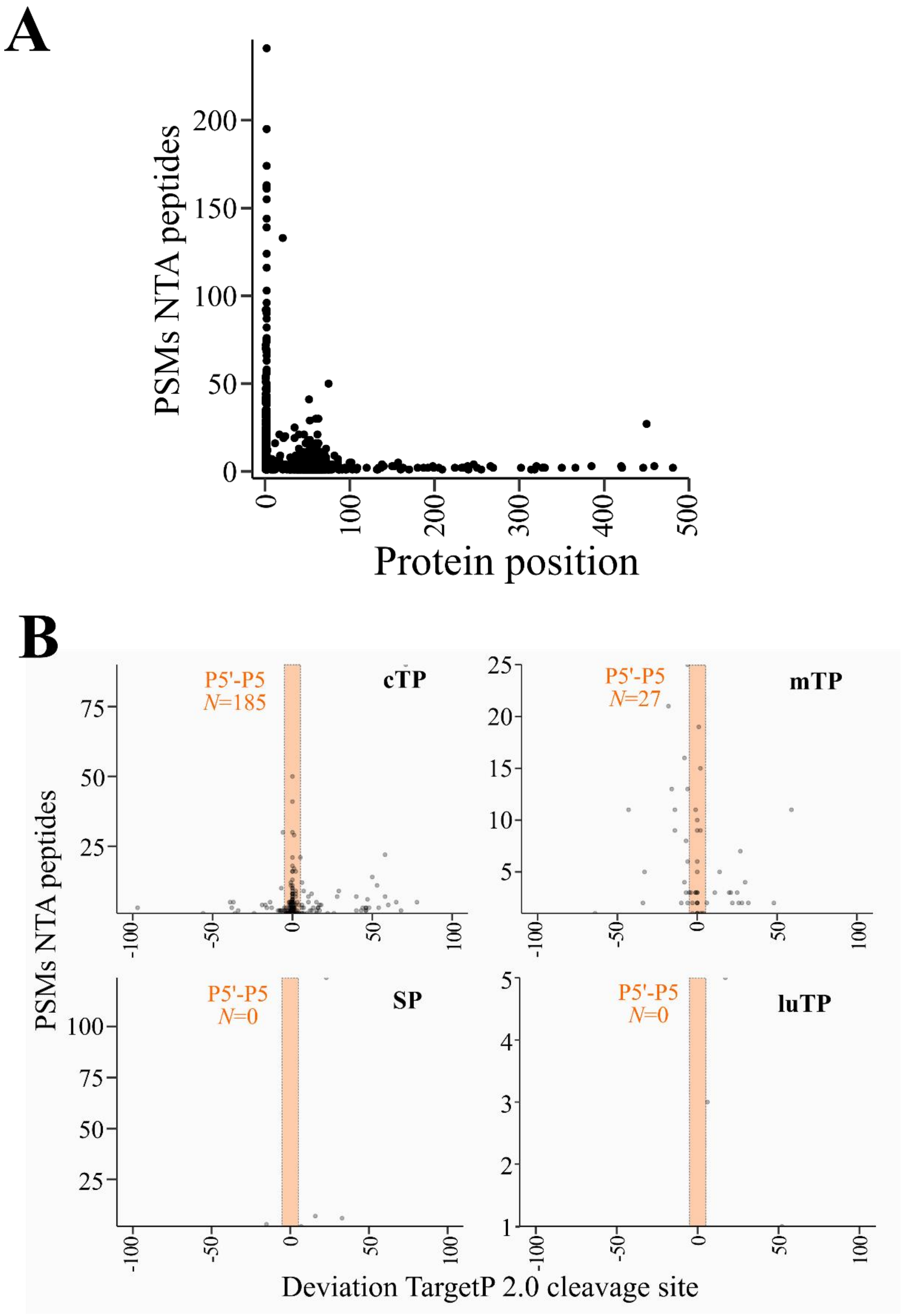

Supplementary Figure 3| Distribution of NTA peptides. **(A)** Number of peptide-to-spectral matches (PSMs) of identified unique NTA Nt peptides in cell cultures in function of protein position (start position of identified NTA Nt peptides were considered). **(B)** PSMs of NTA peptides in cell cultures in function of the deviation to TargetP 2.0 (Almagro Armenteros et al., 2019) predicted P1' cleavage sites for chloroplast transit peptide (cTP), mitochondrial TP (mTP), signal peptide (SP) or luminal TP (luTP). Orange rectangles reflect the P5-P5' predicted regions, considered in this study to assign neo-N-termini indicative of cTP/mTP/SP/luTP cleavage (respective number of neo-N-termini displayed in figure).

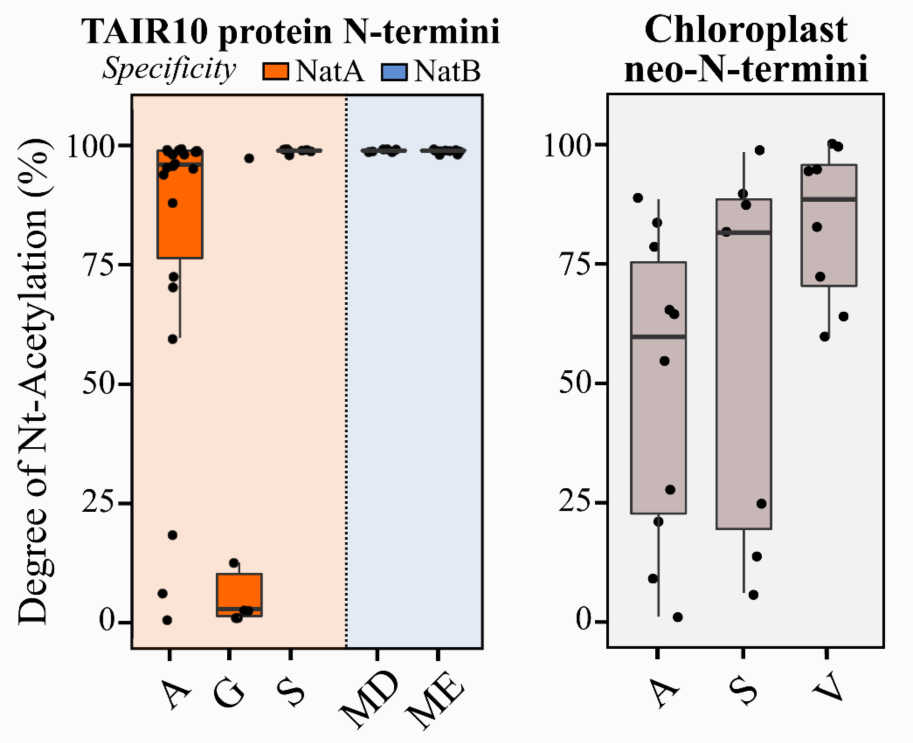

Supplementary Figure 4| The degree of NTA (%) for annotated protein N-termini (*Left*) and chloroplast neo-N-termini (*Right*) were plotted for various types of N-termini according to their NAT specificity profiles. N-termini matching NatA or NatB specificities are indicated in orange and blue, respectively, while chloroplast protein NTA is indicated in grey. Only Nt residue(s) with at least five data points were plotted, for a full overview see **Supplementary Dataset S4**.

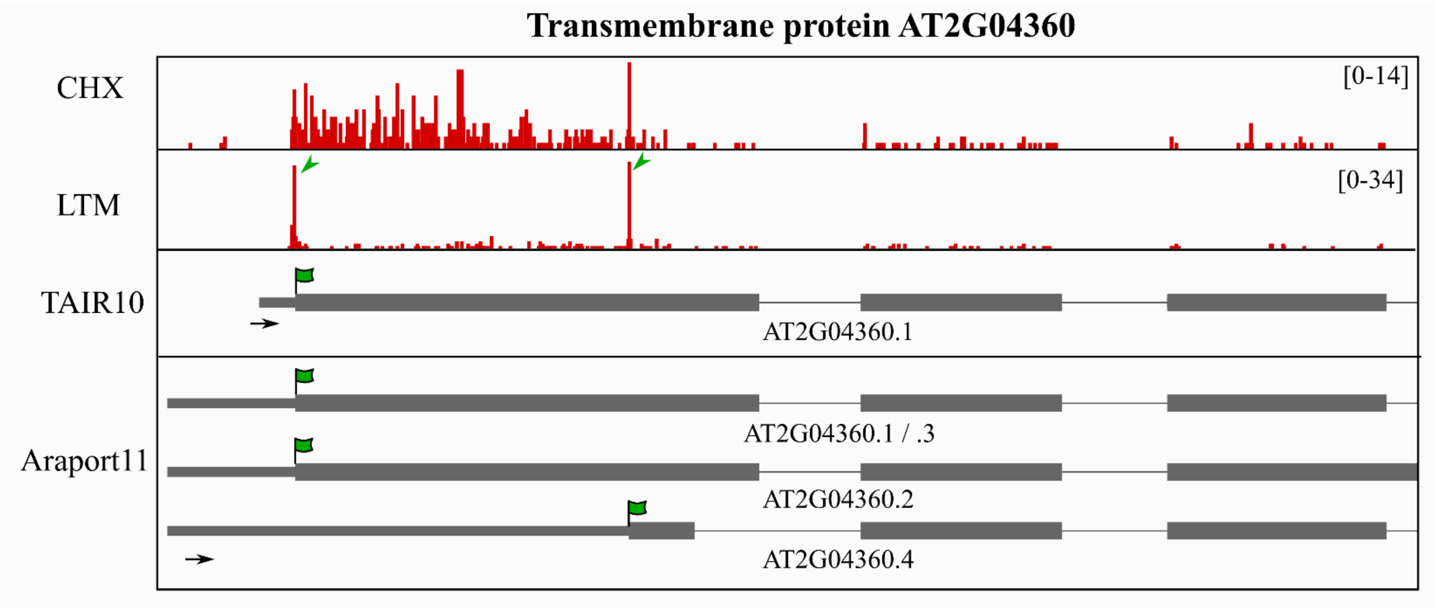

Supplementary Figure 5| Ribo-seq evidence in support of translation initiation at multiple annotated TIS for transmembrane protein AT2G04360. Genome view showing cycloheximide (CHX) and lactimidomycin (LTM) strand-specific positional Ribo-seq data (red). Green flags indicate annotated TAIR10 and/or Araport11 TIS and corresponding LTM peaks were indicated by green arrowheads.

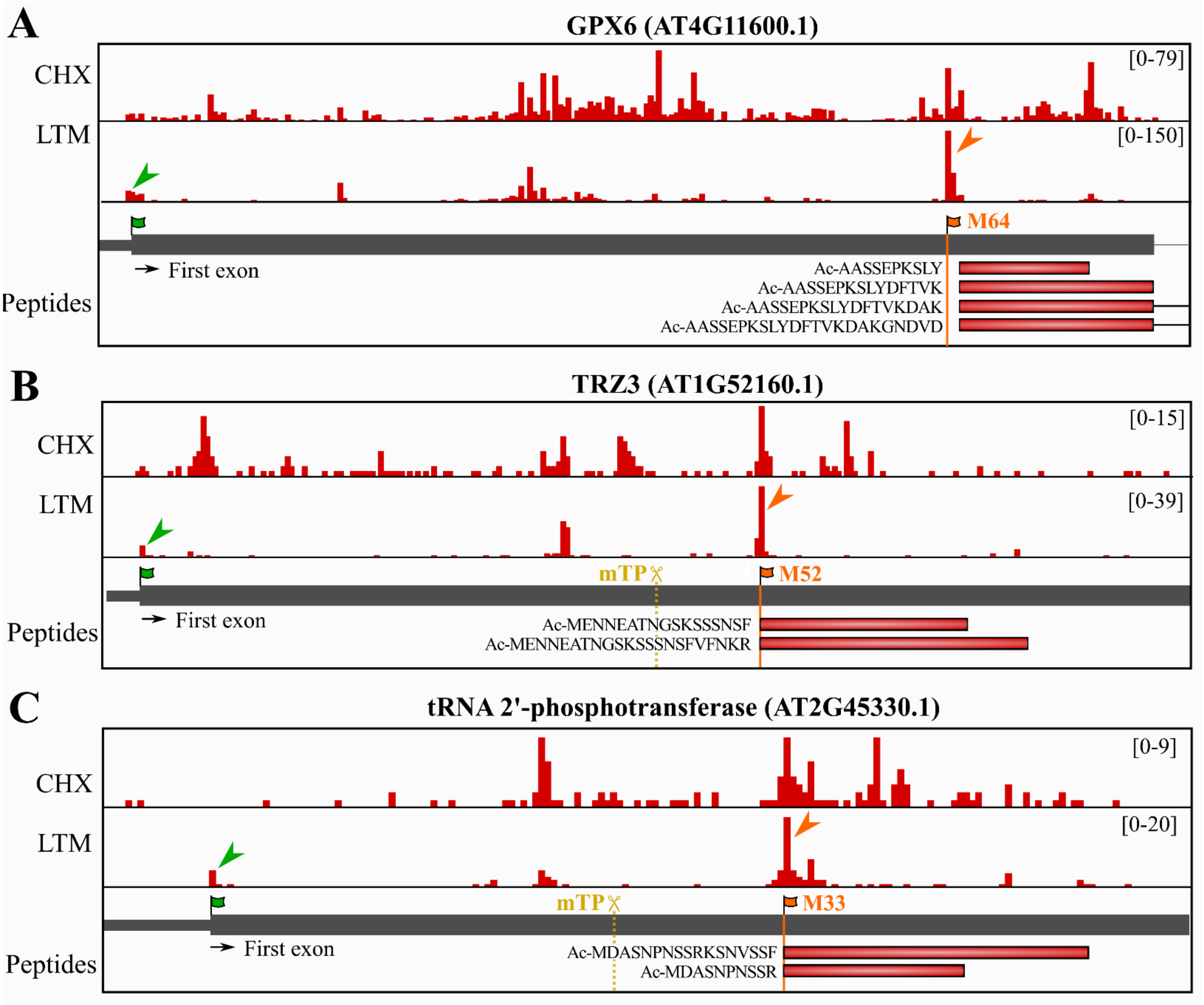

Supplementary Figure 6| Riboproteogenomic identified dTIS of dual localized *GLUTATHIONE PEROXIDASE6* (A), tRNAse Z3 (B) and tRNA 2'-phosphotranferase (C). Genome views showing cycloheximide (CHX) and lactimidomycin (LTM) strand-specific positional Ribo-seq data (red). LTM peaks corresponding to dbTIS and dTIS were indicated by green and orange arrowheads, respectively. Vertical lines indicate identified dTIS (orange) and mTP predicted cleavage site (yellow, dotted line) (Almagro Armenteros et al., 2019). Identified Nt peptide sequences were plotted as red rectangles. In case of GPX6, two Nt peptide sequences span an exon-exon junction.

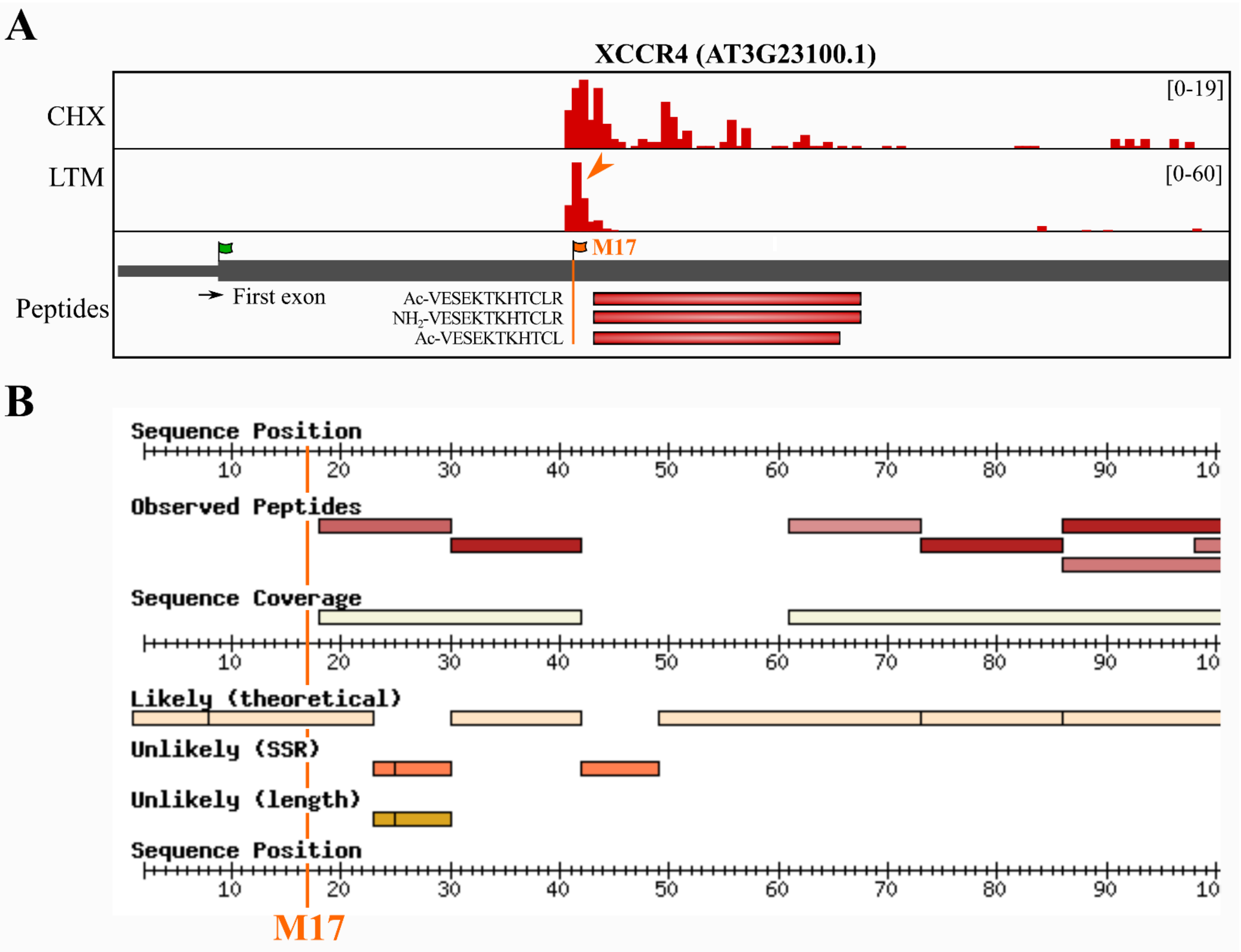

Supplementary Figure 7| (A) Ribo-seq evidence in support of alternative translation initiation at a dTIS corresponding to Met17 of the DNA repair protein homologue *XRCC4* (AT3G23100). Genome view showing cycloheximide (CHX) and lactimidomycin (LTM) strand-specific positional Ribo-seq data (red). Green and orange flags indicate annotated TAIR10 dbTIS and Ribo-seq called dTIS (here corresponding to Met17), respectively. Orange arrowheads indicate LTM peaks matching the dTIS. The vertical orange line indicates the position of the riboproteogenomic-matched dTIS. Nt peptide sequences were plotted as red rectangles. **(B)** Peptide evidence for the N-terminal region of *XRCC4* (position 1 to 100) exported from the *Arabidopsis* PeptideAtlas (http://www.peptideatlas.org/builds/arabidopsis/) (van Wijk et al., 2021).

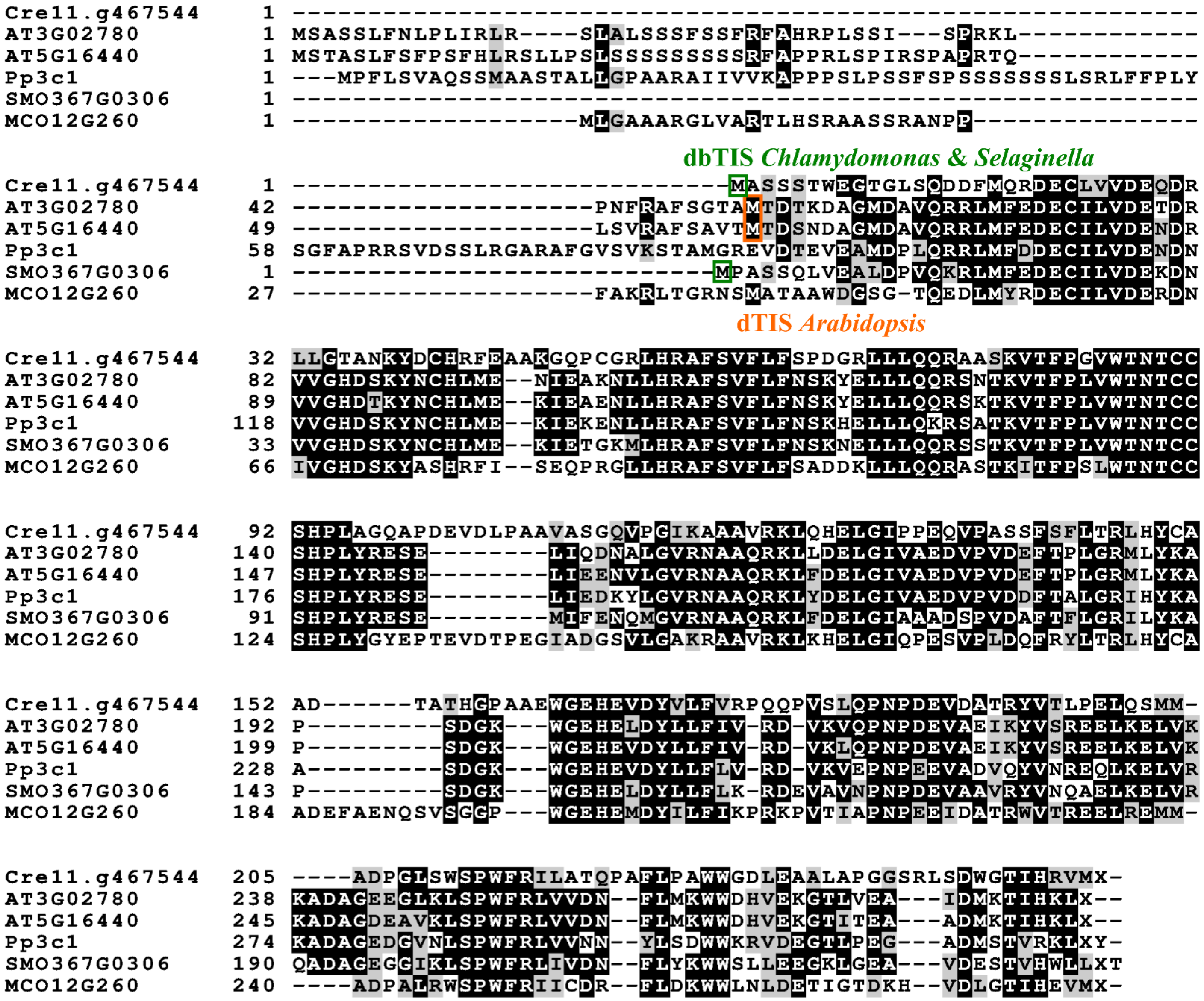

Supplementary Figure 8| Multiple protein sequence alignment of *IPP* homologues. The dTIS identified for *IPP1* (AT5G16440, iMet59) and *IPP2* (AT3G02780, iMet52) were indicated by an orange rectangle. dbTIS of homologs in *Chlamydomonas reinhardtii* (Cre11.g467544) and *Selaginella moellendorffii* (SMO367G0306) were indicated by a green rectangle. Homologous protein sequences were retrieved by PLAZA (Van Bel et al., 2018) (https://bioinformatics.psb.ugent.be/plaza) and protein alignments were constructed by MEGA-X (Kumar et al., 2018) and visualized using the BoxShade tool (https://embnet.vital-it.ch/software/BOX_form.html). Other proteins included in alignment: Pp3c1_33430 (*Physcomitrium patens*) and MCO12G260 (*Micromonas commoda*).

2.2 Supplementary Tables

Supplementary Table 1| Primer pairs used for site-directed mutagenesis PCR (TIS mutation: ATG>TTG) of Ribo-seq identified translation initiation sites in selected genes.

Description	TIS	Sequence forward mutation primer	Sequence reverse mutation primer
Isopentenyl diphosphate isomerase 2 (AT3G02780)	Met1	5'-aaataaagaagaagcagacaaggttatatctccttctggcc-3'	5'-ggccagaaggagatataaccttgtctgcttcttctttattt-3'
	Met52	5'-catctttagtatctgtcaaagcggtaccagagaaagcac-3'	5'-gtgctttctctggtaccgctttgacagatactaaagatg-3'
Plant UBX domain-containing protein 1 (AT3G27310)	Met1	5'-gaagggtcatcaacaaacaaggttatatctccttctggc-3'	5'-gccagaaggagatataaccttgtttgttgatgacccttc-3'
	Met22	5'-tgaagaagaagcttccaaggaatcggtgatctcga-3'	5'-tcgagatcaccgattccttggaagcttcttcttca-3'
NAC domain-containing protein 14 (AT1G33060)	Met1	5'-agttttgtttttatttggttcaaggttatatctccttctggcctt-3'	5'-aaggccagaaggagatataaccttgaaccaaataaaaaacaaaact-3'
	Met12	5'-gcttgctccgtcgtcaactccggtaaagttttg-3'	5'-caaaactttaccggagttgacgacggagcaagc-3'

Supplementary Table 2| Literature supporting translation initiation at Ribo-seq called dTIS identified in this study. Abbreviations: TnT, *in vitro* coupled transcription and translation; TIS, translation initiation site; TSS, transcription start site.

Gene	Description	dTIS	Evidence	Reference
AT5G19150	NAD(P)HX dehydratase	Met45	- TnT mutagenesis<br>- Localized cytosol, plastid and mitochondria	(Niehaus et al., 2014)
AT5G16440	Isopentenyl diphosphate Isomerase 1 (IPP1)	Met59	- Alternative TSS<br>- Localized cytosol and plastid	(Phillips et al., 2008)
AT3G02780	Isopentenyl diphosphate Isomerase 1 (IPP2)	Met52	- Alternative TSS<br>- Localized cytosol and mitochondria	
AT2G47730	Glutathione S-transferase phi 8 (GSTF8)	Met49	- Alternative TSS<br>- Localized cytosol and plastid	(Thatcher et al., 2007)
AT5G27380	Glutathione synthetase 2 (GSH2)	Met62	- Alternative TSS<br>- Localized cytosol and plastid	(Wachter et al., 2005)
AT1G14610	ValRS	Met45	- Leaky scanning<br>- Localized cytosol and mitochondria	(Souciet et al., 1999)
AT5G26830	ThrRS	Met34	- Leaky scanning<br>- Localized cytosol and mitochondria	
AT1G29880	GlyRS	Met40	- Alternative TSS<br>- Localized cytosol and mitochondria	(Duchene et al., 2001)
AT4G37000	Accelerated cell death 2 (ACD2)	Met41	- Mapped dTIS in tomato orthologue (Solyc03g044470)<br>- Orthologue localizes in plastid<br>- dbTIS-mutation localizes in cytosol	(Li and Liu, 2020)
AT4G11600	Glutathione peroxidase 6 (GPX6)	Met64	- Mapped dTIS in *Arabidopsis* seedlings<br>- Localized cytosol and mitochondria	(Liu et al., 2013;Attacha et al., 2017)
AT5G12040	ω-amidase	Met63	- dTIS in human orthologue	(Cooper, 2004;Zhang and Marsolais, 2014)
AT2G30110	Ubiquitin-activating enzyme 1 (UBA1)	Met63	- dTIS in human orthologue	(Stephen et al., 1997)
AT1G52160	tRNAse Z3 (TRZ3)	Met52	- Localized cytosol and mitochondria	(Canino et al., 2009)
AT5G24400	6-phosphogluconolactonase 3 (PGL3)	Met70	- Strong KOZAK sequence<br>- Localized cytosol and peroxisome<br>- But, no influence Met70 mutation to localization	(Holscher et al., 2014)

Supplementary Table 3| Degree of Nt acetylation for Nt peptides matching downstream TIS (dTIS) in the stromal enriched fractions. For each gene locus a description and the respective dTIS position (in the main protein isoform) was given, as well as the predicted Target 2.0 cleavage site (Almagro Armenteros et al., 2019) indicated in bold when matching the identified Nt peptide start position. The NTA N-terminus was displayed with its degree of N-terminal acetylation (%NTA) in stromal fractions. dTIS were listed based on decreasing % of NTA. For more details see Supplementary Dataset S4.

Gene	Description	dTIS	TargetP 2.0	N-terminus	%NTA
AT1G29880	Gly-tRNA synthetase	Met40	Pos 22 (mTP)	Ace-MD	100%
AT3G27310	Plant UBX domain-containing protein 1 (PUX1)	Met22	noTP	Ace-ME	100%
AT1G49480	Related to vernalization1 1 (RTV1)	Met3	noTP	Ace-MD	100%
AT5G36700	2-Phosphoglycolate phosphatase 1 (PGLP1)	Met54	Pos 65 (cTP)	Ace-T	99%
AT1G66240	Homolog of anti-oxidant 1 (ATX1)	Met31	noTP	Ace-S	99%
AT3G02780	Isopentenyl diphosphate isomerase 2 (IPP2)	Met52	**Pos 53 (cTP)**	Ace-T	95%
AT5G16440	Isopentenyl diphosphate isomerase 1 (IPP1)	Met59	Pos 54 (cTP)	Ace-T	91%
AT3G47590	Alpha/beta-Hydrolases superfamily protein	Met50	noTP	Ace-MD	88%
AT5G12040	ω-amidase	Met63	**Pos 64 (cTP)**	Ace-A	76%
AT1G55805	BolA-like family protein	Met52	Pos 37 (mTP)	Ace-S	27%
AT5G19150	NAD(P)HX dehydratase	Met45	**Pos 46 (mTP)**	Ace-S	36%

References

Almagro Armenteros, J.J., Salvatore, M., Emanuelsson, O., Winther, O., Von Heijne, G., Elofsson, A., and Nielsen, H. (2019). Detecting sequence signals in targeting peptides using deep learning. *Life Sci Alliance* 2.

Attacha, S., Solbach, D., Bela, K., Moseler, A., Wagner, S., Schwarzlander, M., Aller, I., Muller, S.J., and Meyer, A.J. (2017). Glutathione peroxidase-like enzymes cover five distinct cell compartments and membrane surfaces in Arabidopsis thaliana. *Plant Cell Environ* 40**,** 1281-1295.

Canino, G., Bocian, E., Barbezier, N., Echeverria, M., Forner, J., Binder, S., and Marchfelder, A. (2009). Arabidopsis encodes four tRNase Z enzymes. *Plant Physiol* 150**,** 1494-1502.

Cooper, A.J. (2004). The role of glutamine transaminase K (GTK) in sulfur and alpha-keto acid metabolism in the brain, and in the possible bioactivation of neurotoxicants. *Neurochem Int* 44**,** 557-577.

Duchene, A.M., Peeters, N., Dietrich, A., Cosset, A., Small, I.D., and Wintz, H. (2001). Overlapping destinations for two dual targeted glycyl-tRNA synthetases in Arabidopsis thaliana and Phaseolus vulgaris. *J Biol Chem* 276**,** 15275-15283.

Holscher, C., Meyer, T., and Von Schaewen, A. (2014). Dual-targeting of Arabidopsis 6-phosphogluconolactonase 3 (PGL3) to chloroplasts and peroxisomes involves interaction with Trx m2 in the cytosol. *Mol Plant* 7**,** 252-255.

Kumar, S., Stecher, G., Li, M., Knyaz, C., and Tamura, K. (2018). MEGA X: Molecular Evolutionary Genetics Analysis across Computing Platforms. *Mol Biol Evol* 35**,** 1547-1549.

Li, Y.R., and Liu, M.J. (2020). Prevalence of alternative AUG and non-AUG translation initiators and their regulatory effects across plants. *Genome Res* 30**,** 1418-1433.

Liu, M.J., Wu, S.H., Wu, J.F., Lin, W.D., Wu, Y.C., Tsai, T.Y., Tsai, H.L., and Wu, S.H. (2013). Translational landscape of photomorphogenic Arabidopsis. *Plant Cell* 25**,** 3699-3710.

Niehaus, T.D., Richardson, L.G., Gidda, S.K., Elbadawi-Sidhu, M., Meissen, J.K., Mullen, R.T., Fiehn, O., and Hanson, A.D. (2014). Plants utilize a highly conserved system for repair of NADH and NADPH hydrates. *Plant Physiol* 165**,** 52-61.

Phillips, M.A., D'auria, J.C., Gershenzon, J., and Pichersky, E. (2008). The Arabidopsis thaliana type I Isopentenyl Diphosphate Isomerases are targeted to multiple subcellular compartments and have overlapping functions in isoprenoid biosynthesis. *Plant Cell* 20**,** 677-696.

Souciet, G., Menand, B., Ovesna, J., Cosset, A., Dietrich, A., and Wintz, H. (1999). Characterization of two bifunctional Arabdopsis thaliana genes coding for mitochondrial and cytosolic forms of valyl-tRNA synthetase and threonyl-tRNA synthetase by alternative use of two in-frame AUGs. *Eur J Biochem* 266**,** 848-854.

Stephen, A.G., Trausch-Azar, J.S., Handley-Gearhart, P.M., Ciechanover, A., and Schwartz, A.L. (1997). Identification of a region within the ubiquitin-activating enzyme required for nuclear targeting and phosphorylation. *J Biol Chem* 272**,** 10895-10903.

Thatcher, L.F., Carrie, C., Andersson, C.R., Sivasithamparam, K., Whelan, J., and Singh, K.B. (2007). Differential gene expression and subcellular targeting of Arabidopsis glutathione S-transferase F8 is achieved through alternative transcription start sites. *J Biol Chem* 282**,** 28915-28928.

Van Bel, M., Diels, T., Vancaester, E., Kreft, L., Botzki, A., Van De Peer, Y., Coppens, F., and Vandepoele, K. (2018). PLAZA 4.0: an integrative resource for functional, evolutionary and comparative plant genomics. *Nucleic Acids Res* 46**,** D1190-D1196.

Wachter, A., Wolf, S., Steininger, H., Bogs, J., and Rausch, T. (2005). Differential targeting of GSH1 and GSH2 is achieved by multiple transcription initiation: implications for the compartmentation of glutathione biosynthesis in the Brassicaceae. *Plant J* 41**,** 15-30.

Zhang, Q., and Marsolais, F. (2014). Identification and characterization of omega-amidase as an enzyme metabolically linked to asparagine transamination in Arabidopsis. *Phytochemistry* 99**,** 36-43.
